# Supplementary figures and images for: Elucidation of independently modulated genes in Streptococcus pyogenes reveals carbon sources that control its expression of hemolytic toxins
Source: mSystems. 2023 Jun 6;8(3):e00247-23. doi: 10.1128/msystems.00247-23 (PMC10308926; doi:10.1128/msystems.00247-23)

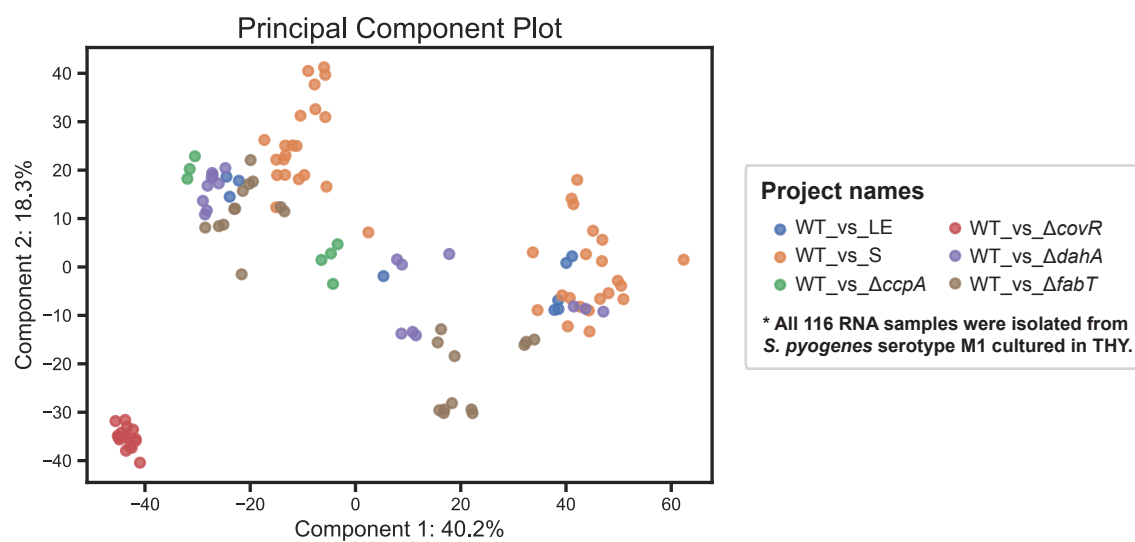

Supplement: FIG S1 — Diversity of 116 RNA-seq data sets used for ICA decomposition. Loadings of the first two principal components (PC). The variation in locations across 116 RNA-seq samples demonstrates the diversity of the compendium. [file msystems.00247-23-s0001.pdf]

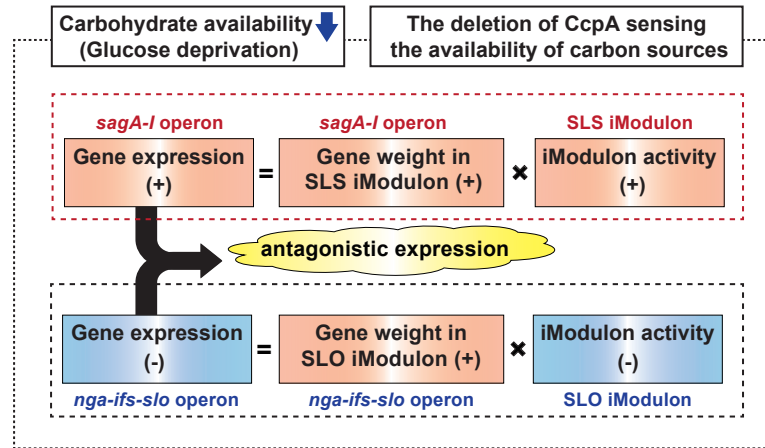

Supplement: FIG S2 — Conditions that S. pyogenes may show the antagonistic expression of sag A-I and nga-ifs-slo operons. [file msystems.00247-23-s0002.pdf]

**A**  $\Delta malR2$  / WT  
in mid-exponential phase

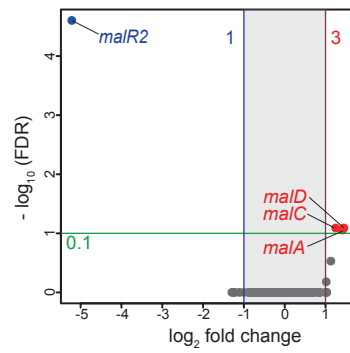

**B**  $\Delta malR2$  / WT  
in stationary phase

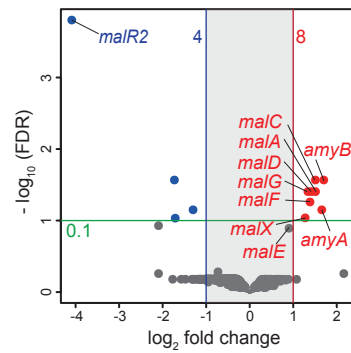

Supplement: FIG S3 — The actual MalR2 regulon. Differentially expressed genes (DEGs) from comparisons of the ΔmalR2 mutant and WT strains at (A) mid-exponential, and (B) stationary growth phases in THY broth. Colored circles indicate significantly upregulated (red) and downregulated (blue) genes (absolute log2 fold change, > 1; adjusted P < 0.1). [file msystems.00247-23-s0003.pdf]
