# Supplementary material for: Elucidation of independently modulated genes in Streptococcus pyogenes reveals carbon sources that control its expression of hemolytic toxins
Source: mSystems. 2023 Jun 6;8(3):e00247-23. doi: 10.1128/msystems.00247-23 (PMC10308926; doi:10.1128/msystems.00247-23)

FabT iModulon

Significantly overlapped TRN: FabT > LacD.1_M14

Biological function: Fatty acid biosynthesis

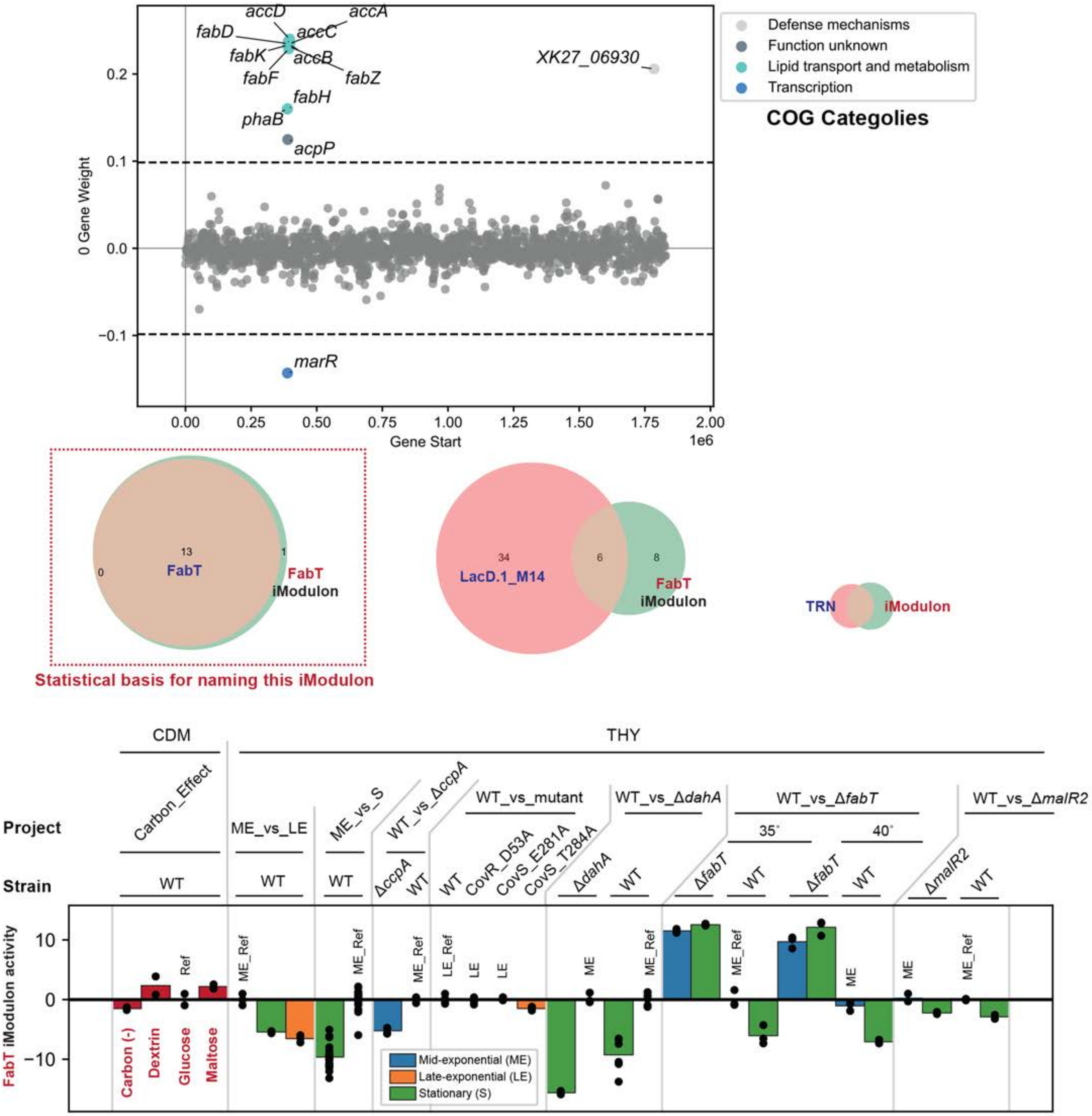

CovR-1 iModulon

Significantly overlapped TRN:

CovR_1_ME_M1 > Mga_low_Glucose_M1 > CovR_CcpA_M1 >Rgg_exponetional_M49 > CodY_M49

Biological function: Phathogenesis and the peptide transport

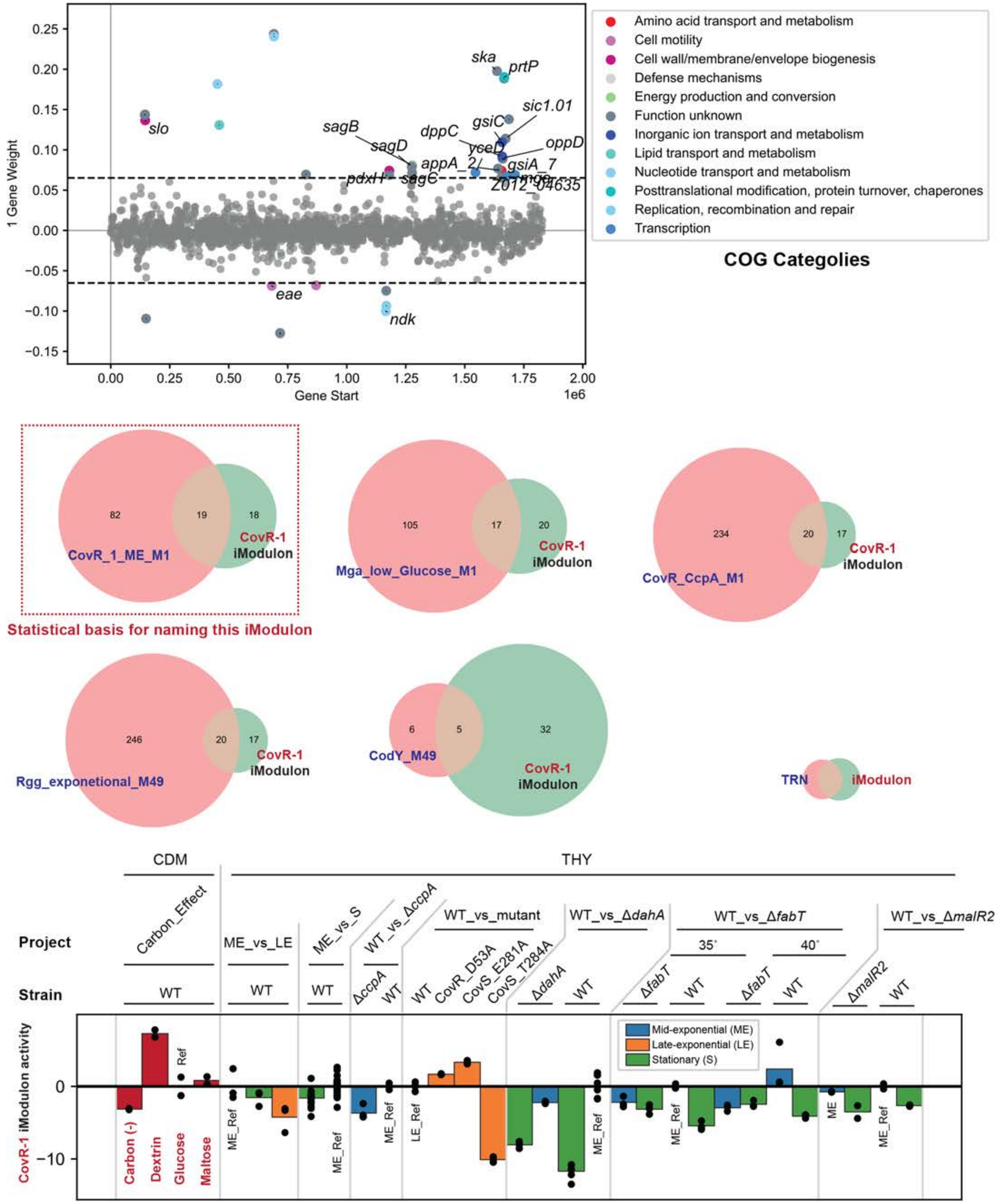

Phage_1-1 iModulon

Significantly overlapped TRN: Phage_1 > SPy1285

Biological function: Prophages

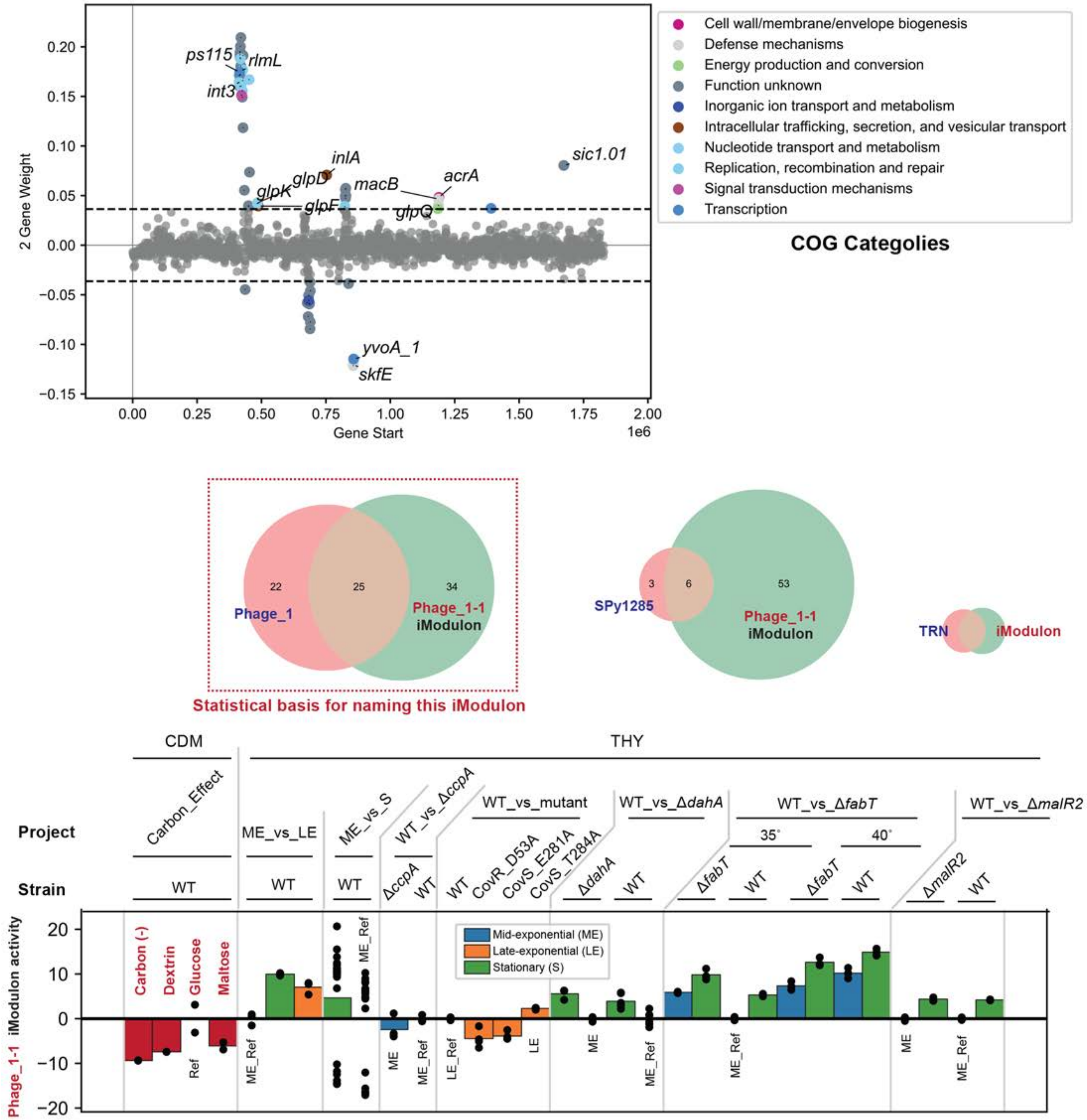

Unc_1 iModulon

Significantly overlapped TRN: ---

Biological function: Carbon use

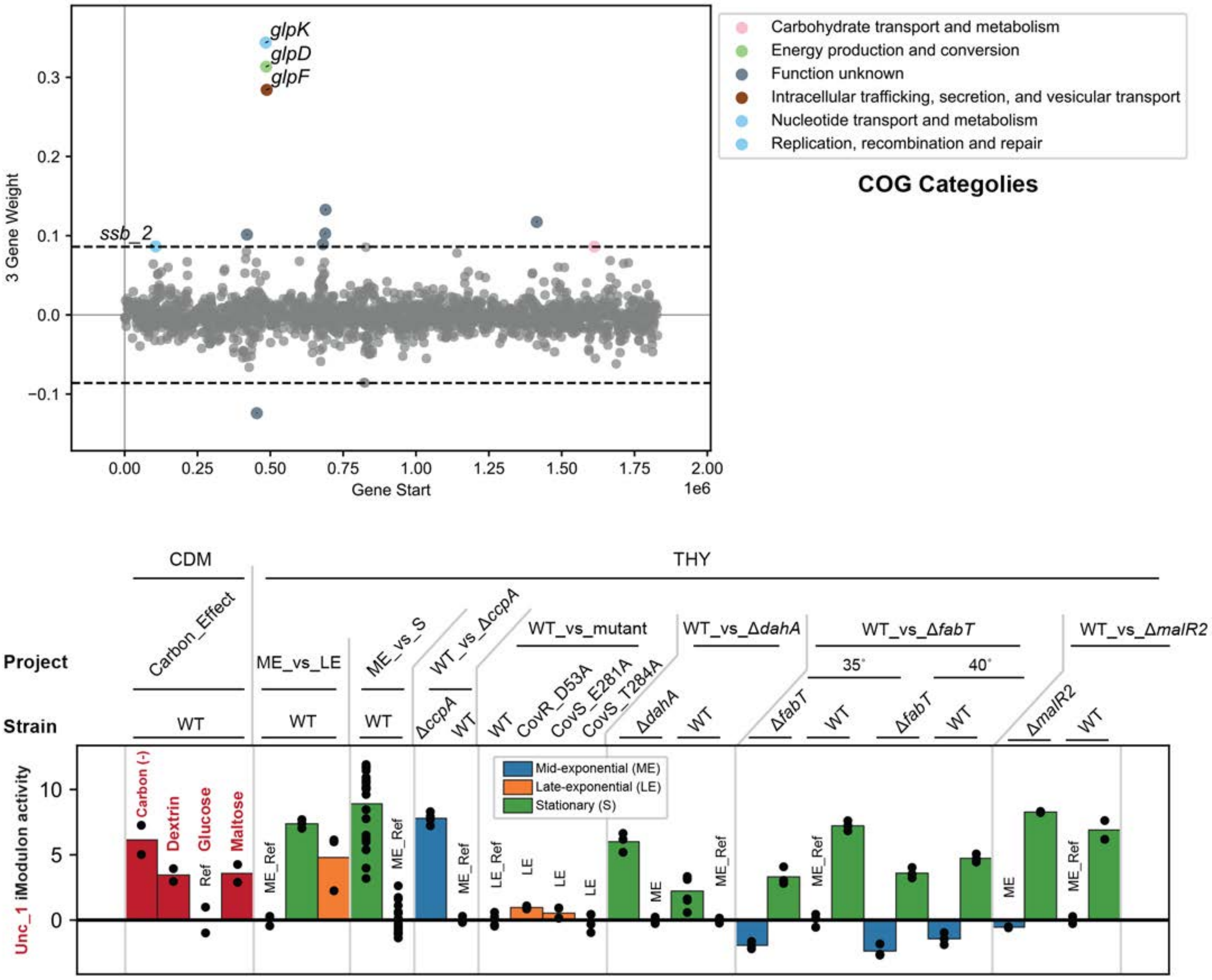

GlnR iModulon

Significantly overlapped TRN: GlnR

Biological function: Amino acid use

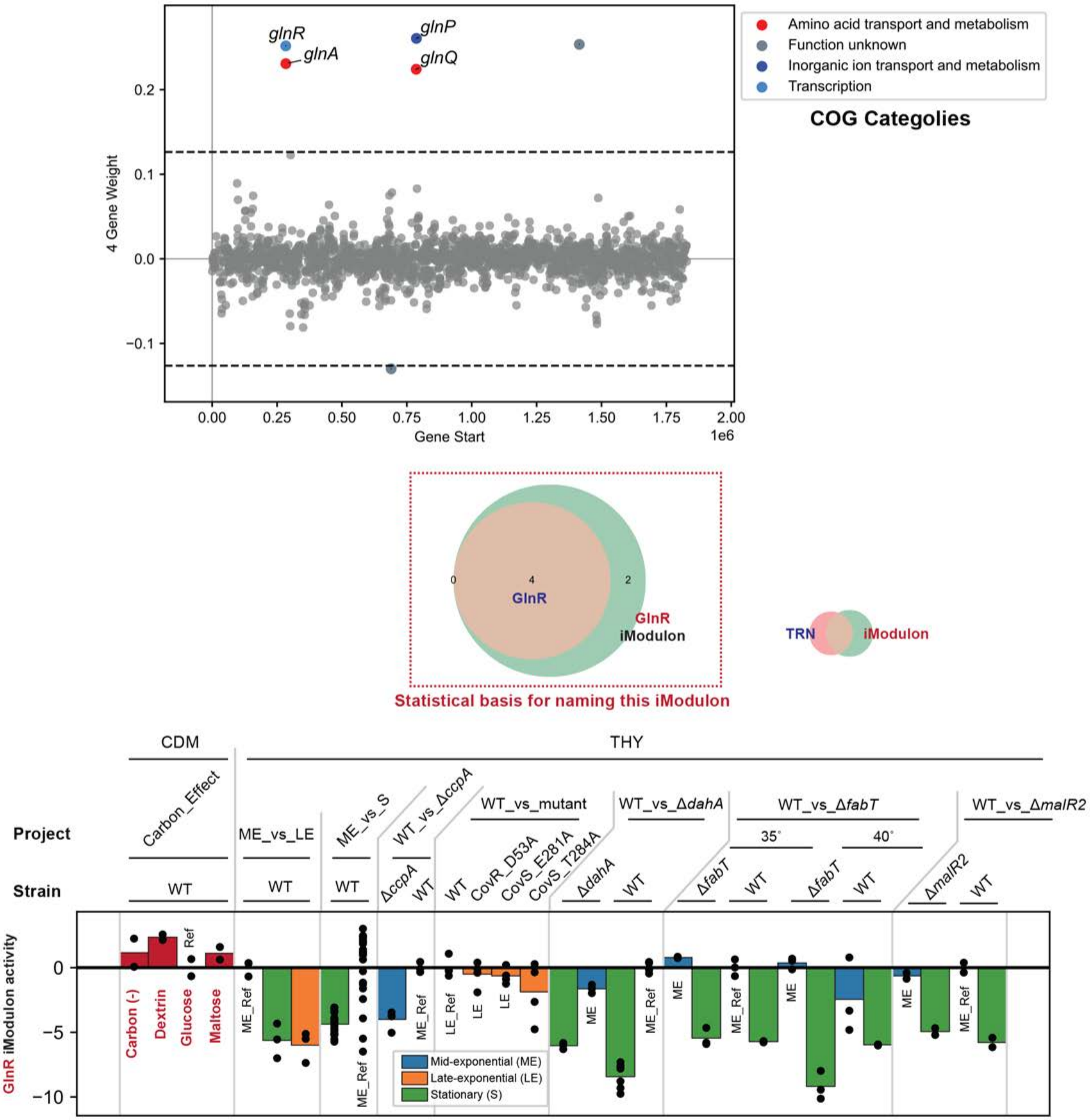

CcpA-1 iModulon

Significantly overlapped TRN: CcpA_core_M1 > CovR_2_LE_M1

Biological function: Amino acid use

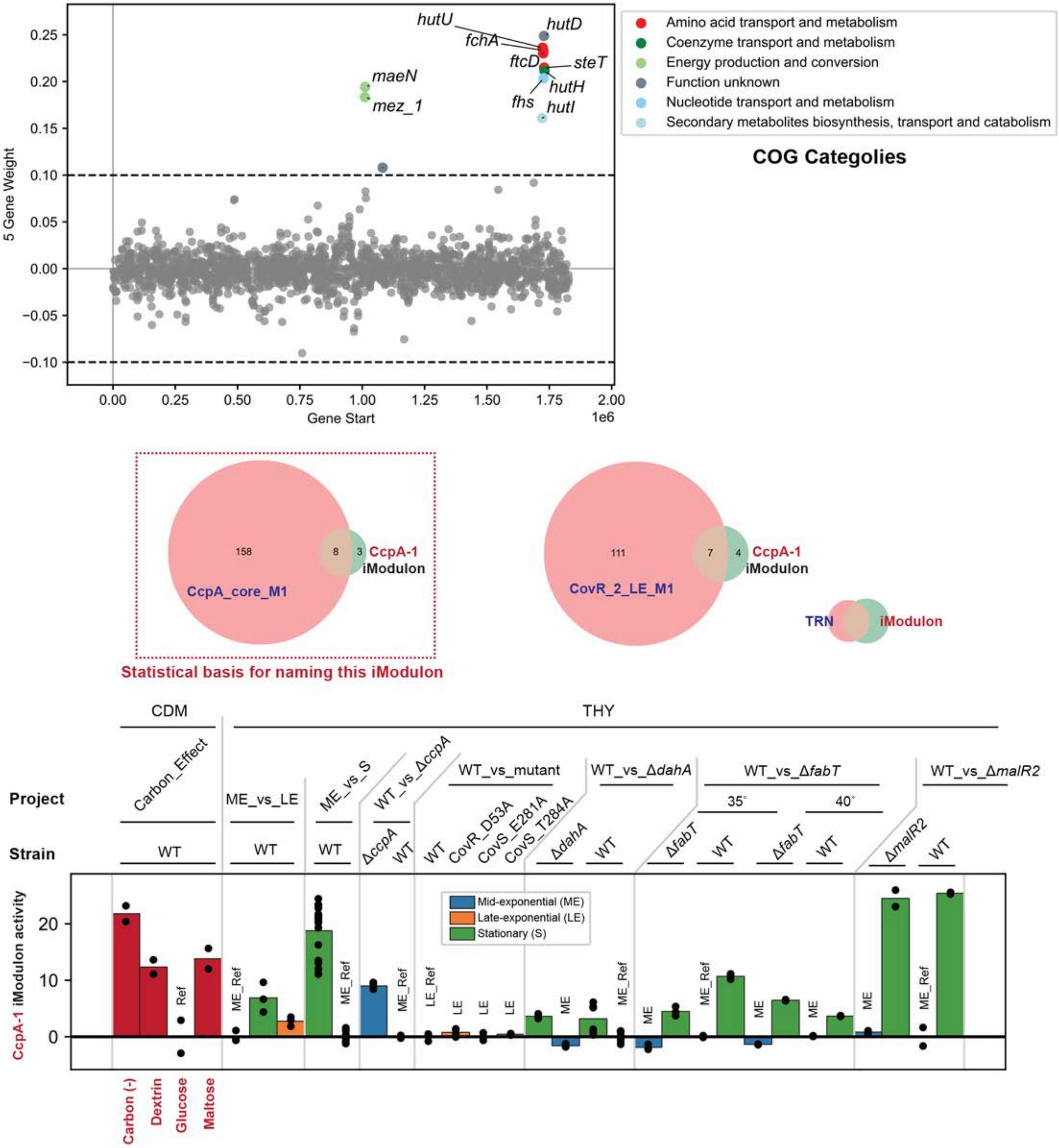

Rgg iModulon

Significantly overlapped TRN: Rgg_exponetional_M49 > CcpA_core_M1 > CovR_CcpA_M1

Biological function: Multi-purpose metabolism

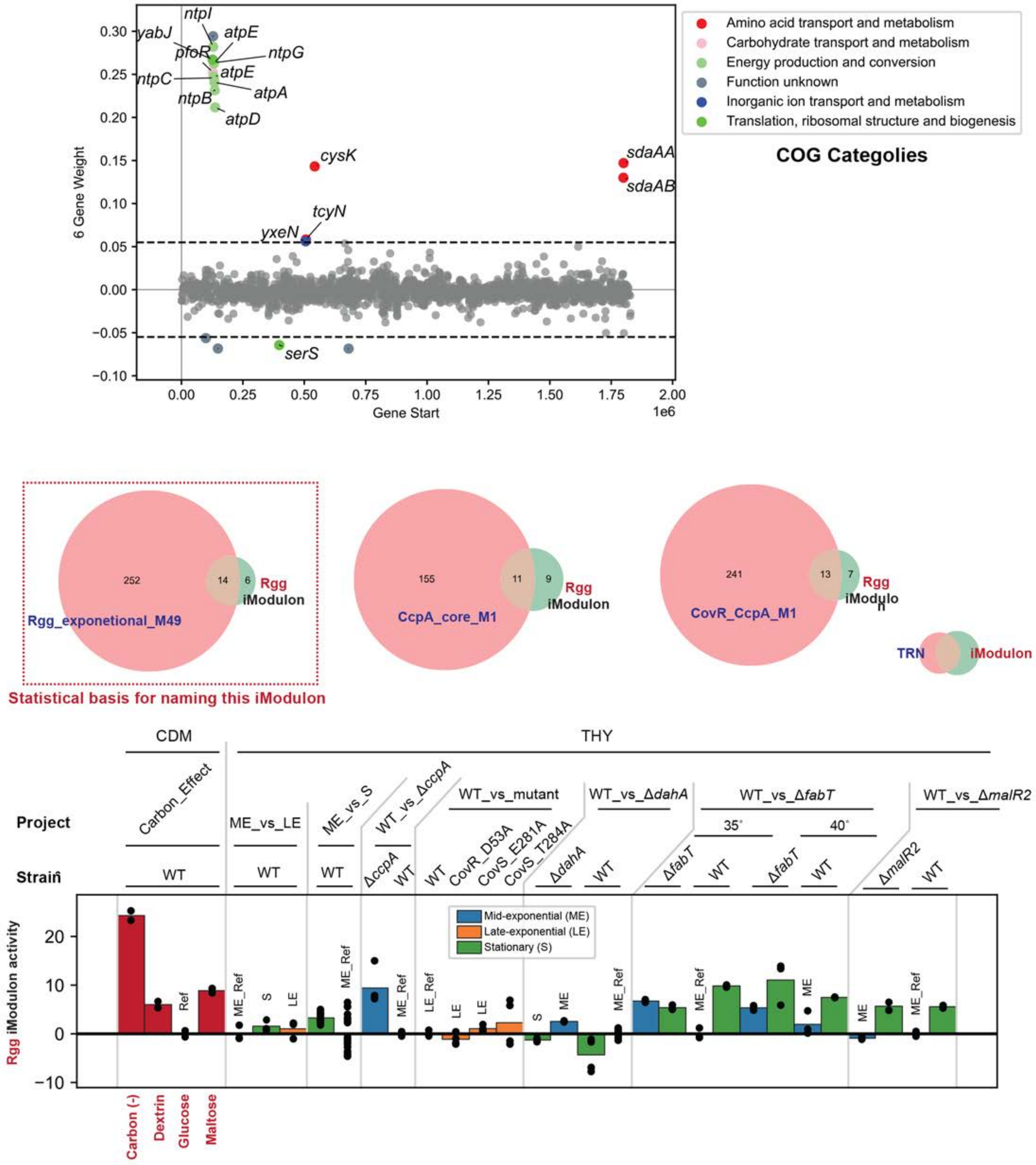

Phage_2-1 iModulon

Significantly overlapped TRN: Phage_2

Biological function: Prophages

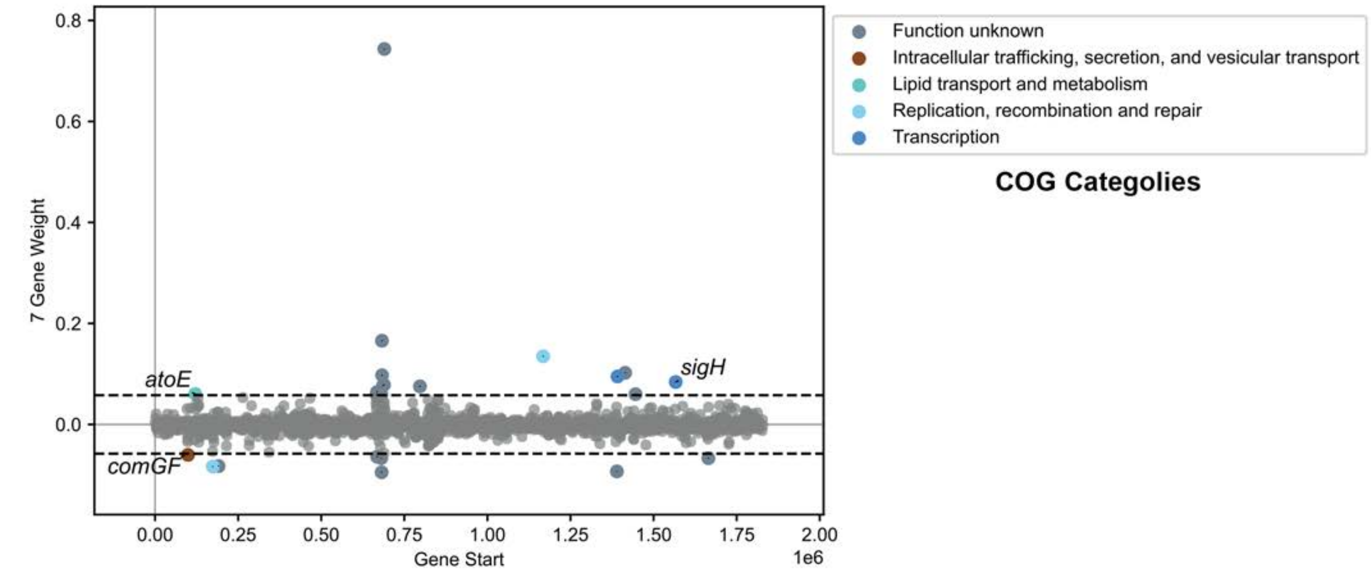

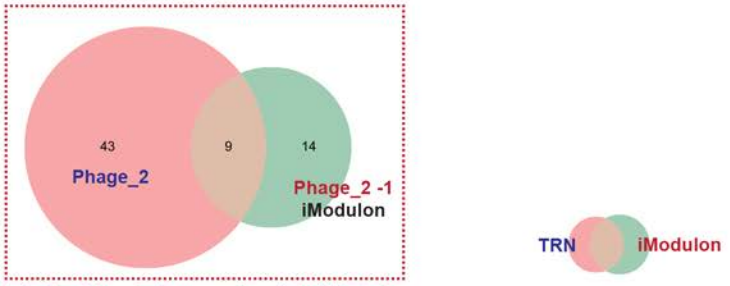

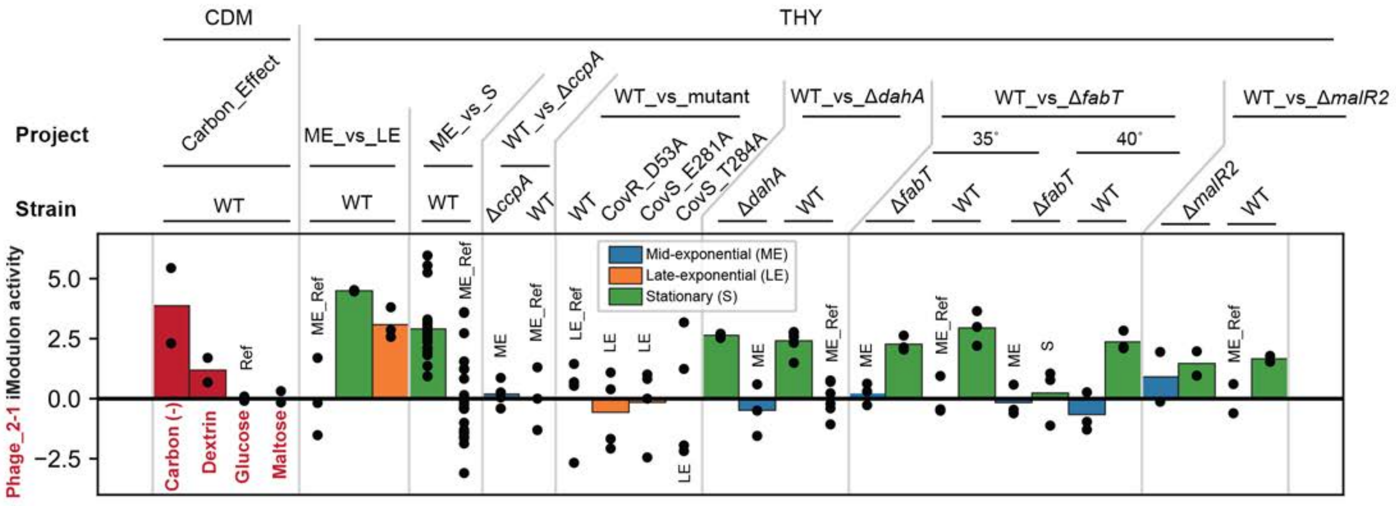

PyrR iModulon

Significantly overlapped TRN: PyrR > MtsR_M49 > Rgg_exponetional_M49 > CovR_1_S_M1

Biological function: Nucleotide use

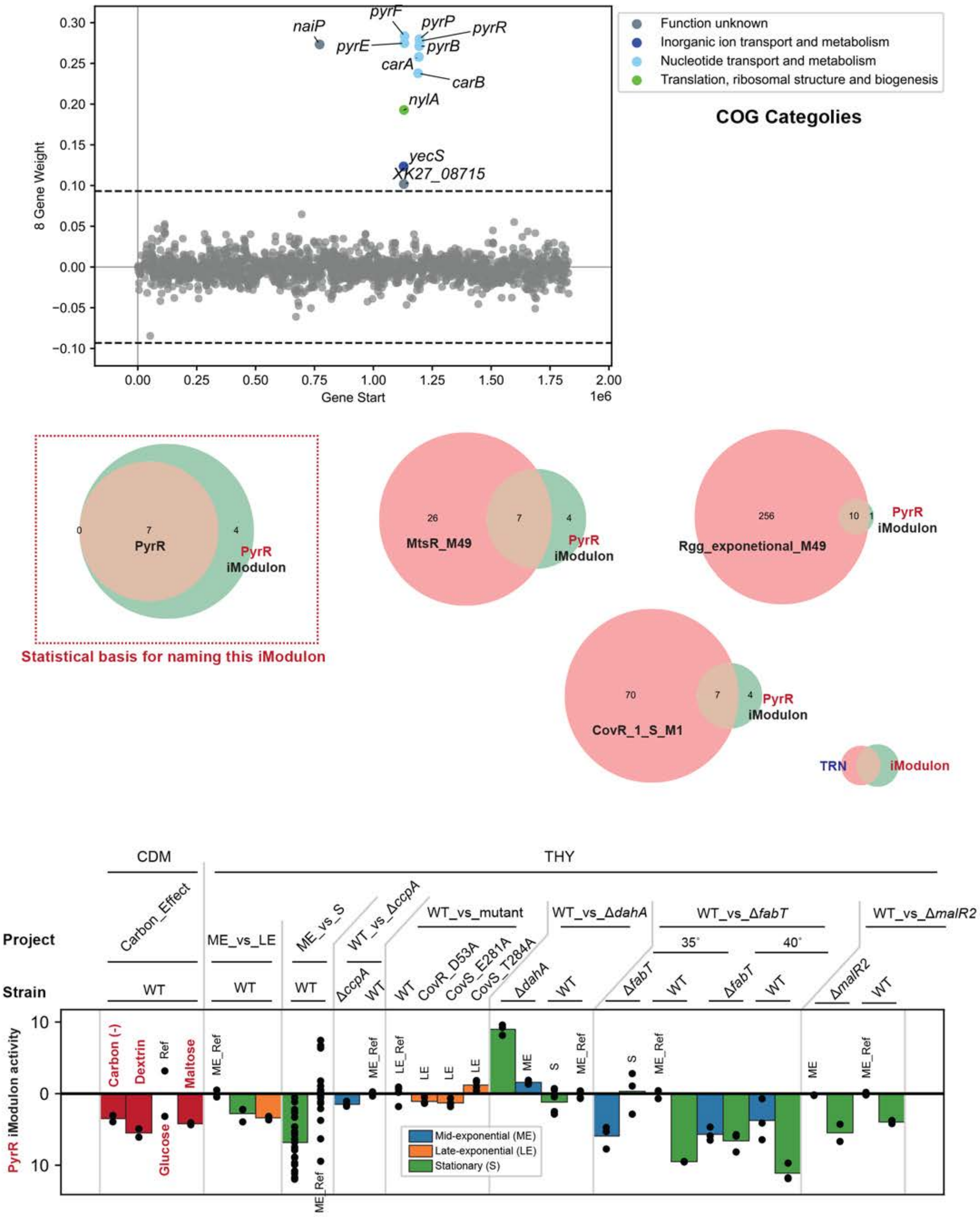

MalR iModulon

Significantly overlapped TRN: MalR_M1 > MalR > CcpA > FruR

Biological function: Carbon use

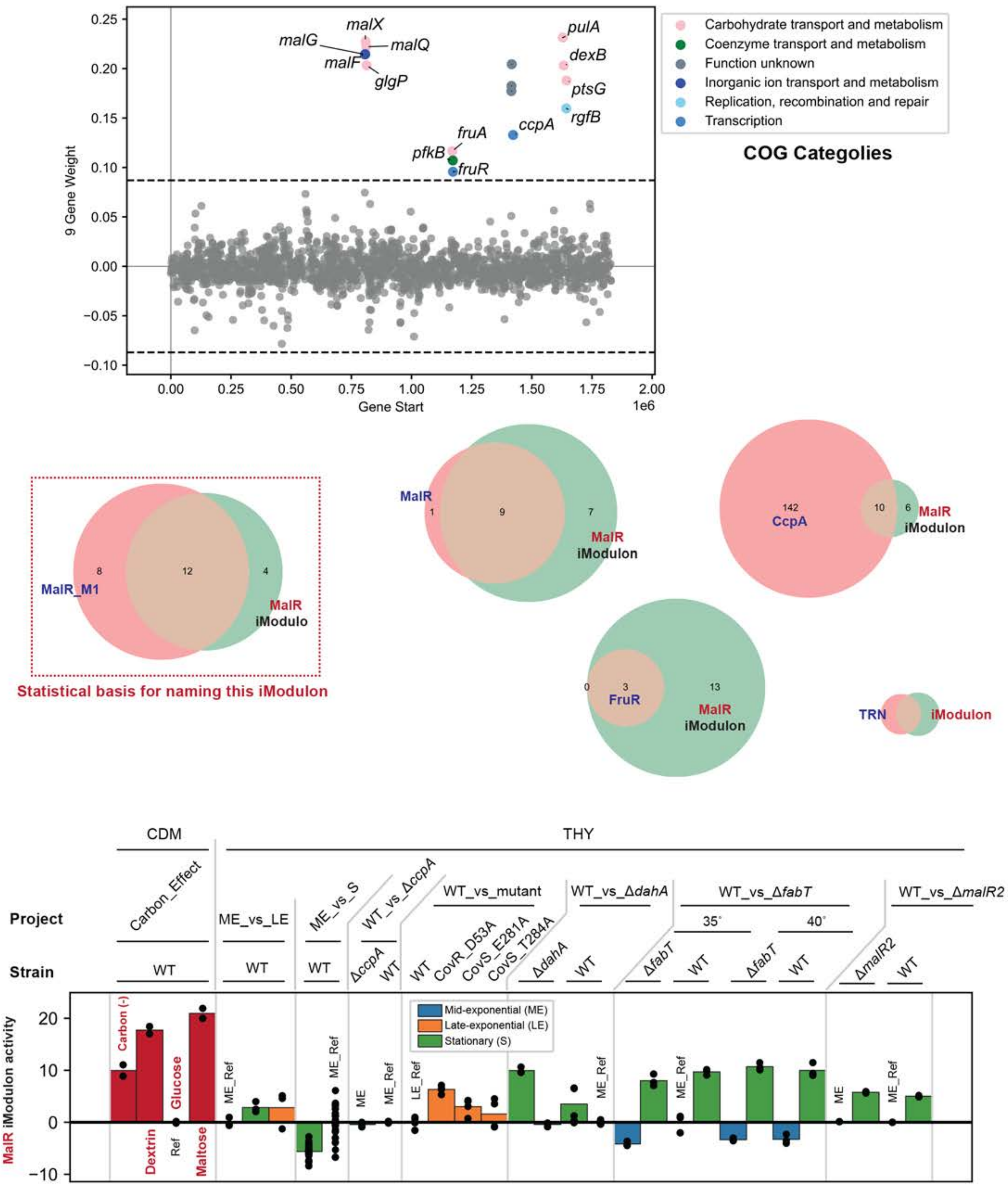

Unc_2 iModulon

Significantly overlapped TRN: ---

Biological function: ---

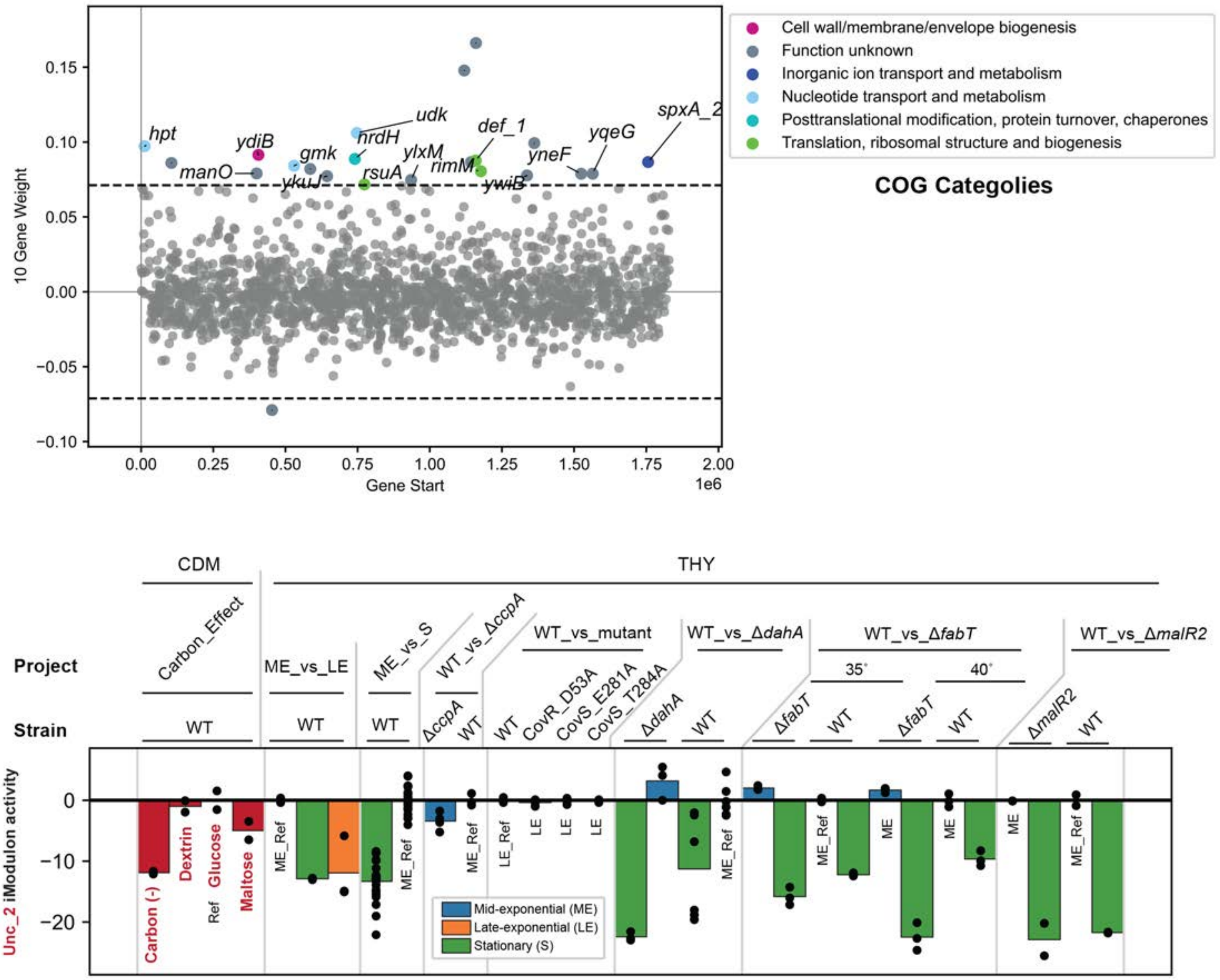

Unc_3 iModulon

Significantly overlapped TRN: ---

Biological function: Translation

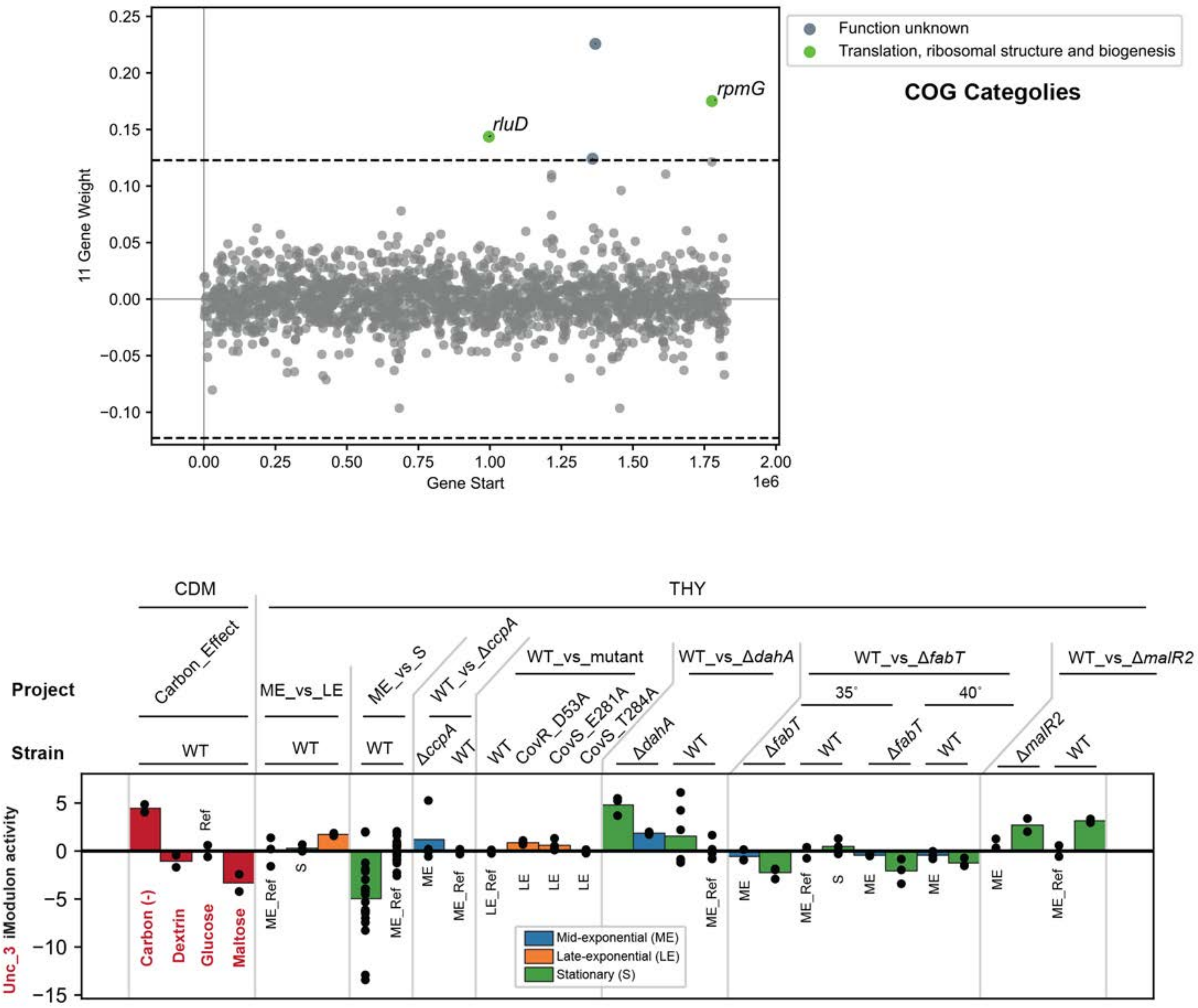

VicR iModulon

Significantly overlapped TRN: VicR

Biological function: Carbon use

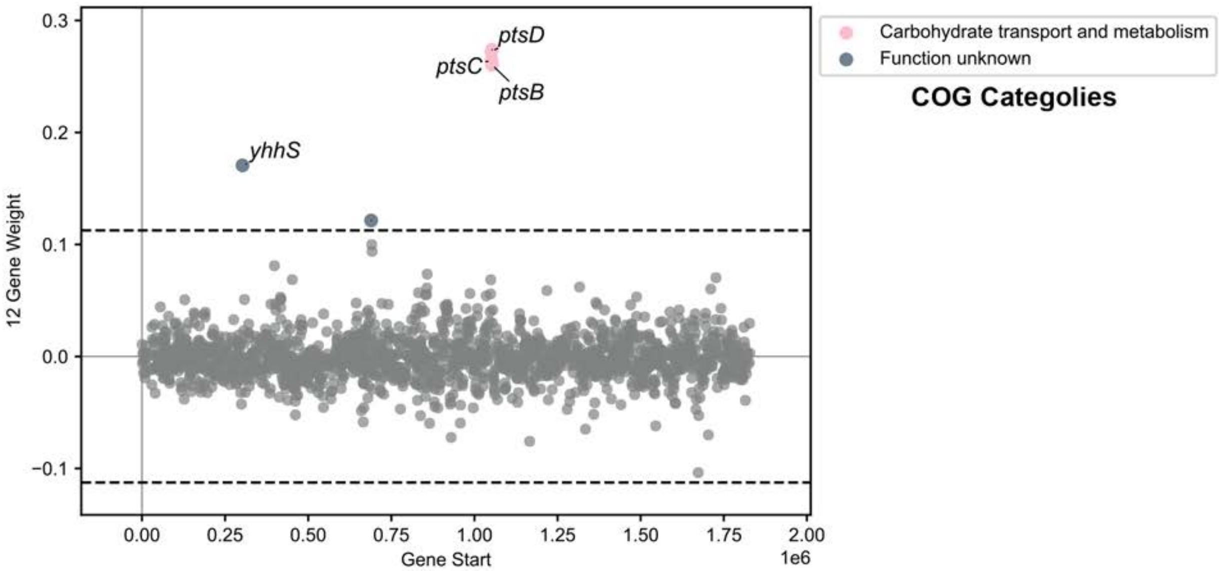

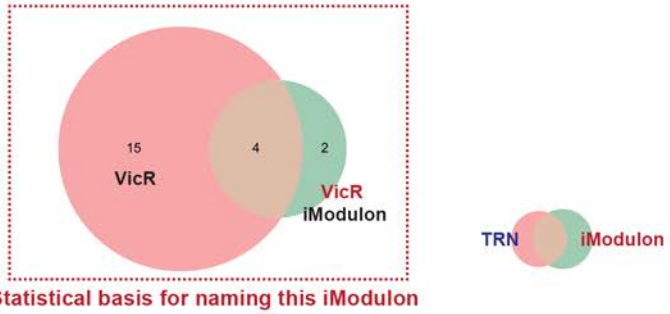

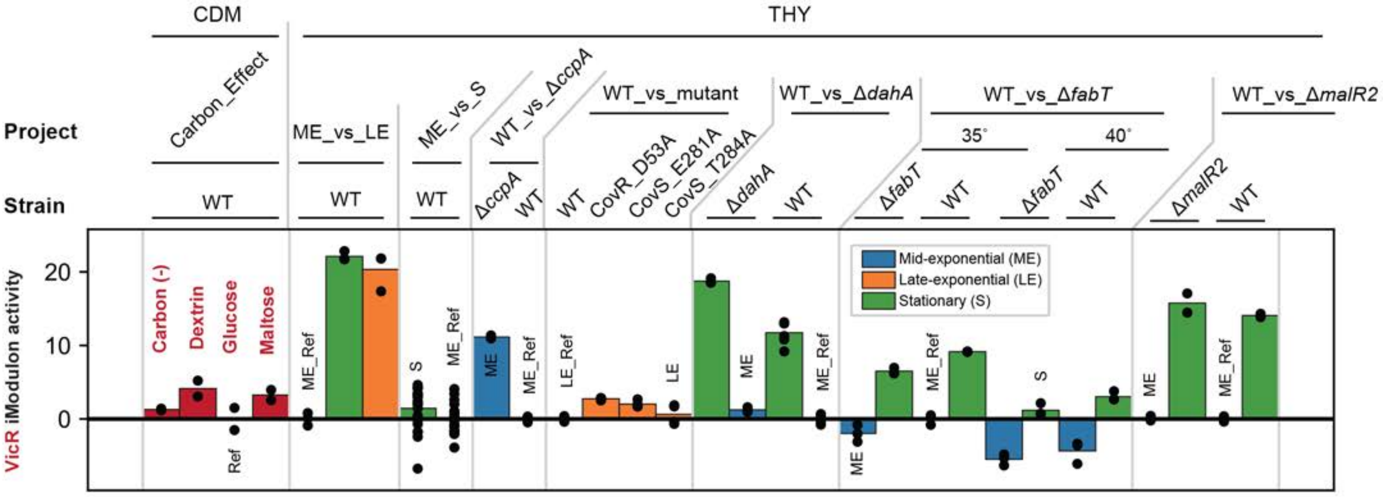

Phage_1-2 iModulon

Significantly overlapped TRN: Phage_1 > SPy1285

Biological function: Prophages

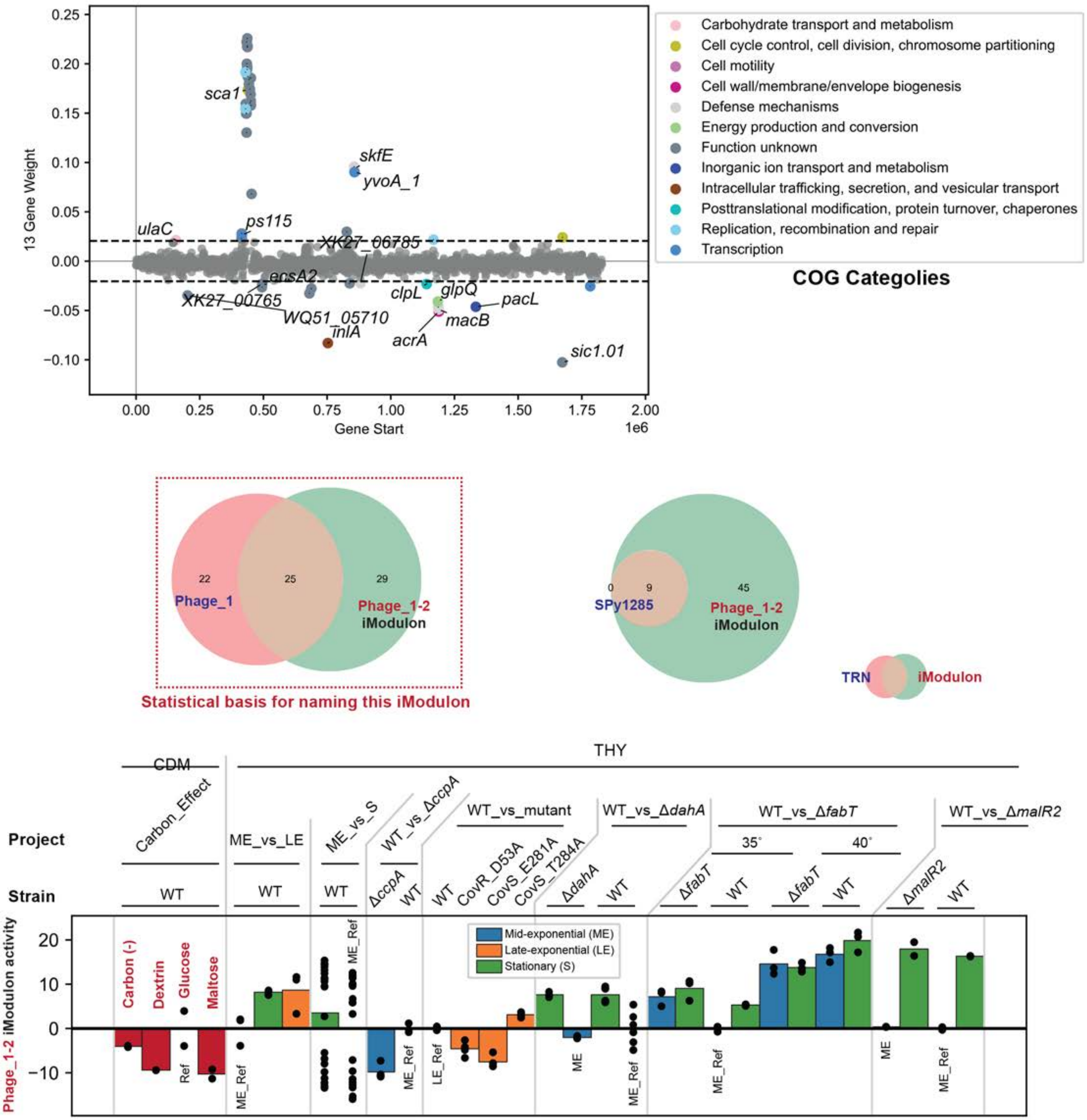

Unc_4 iModulon

Significantly overlapped TRN: ---

Biological function: Multi-purpose metabolism

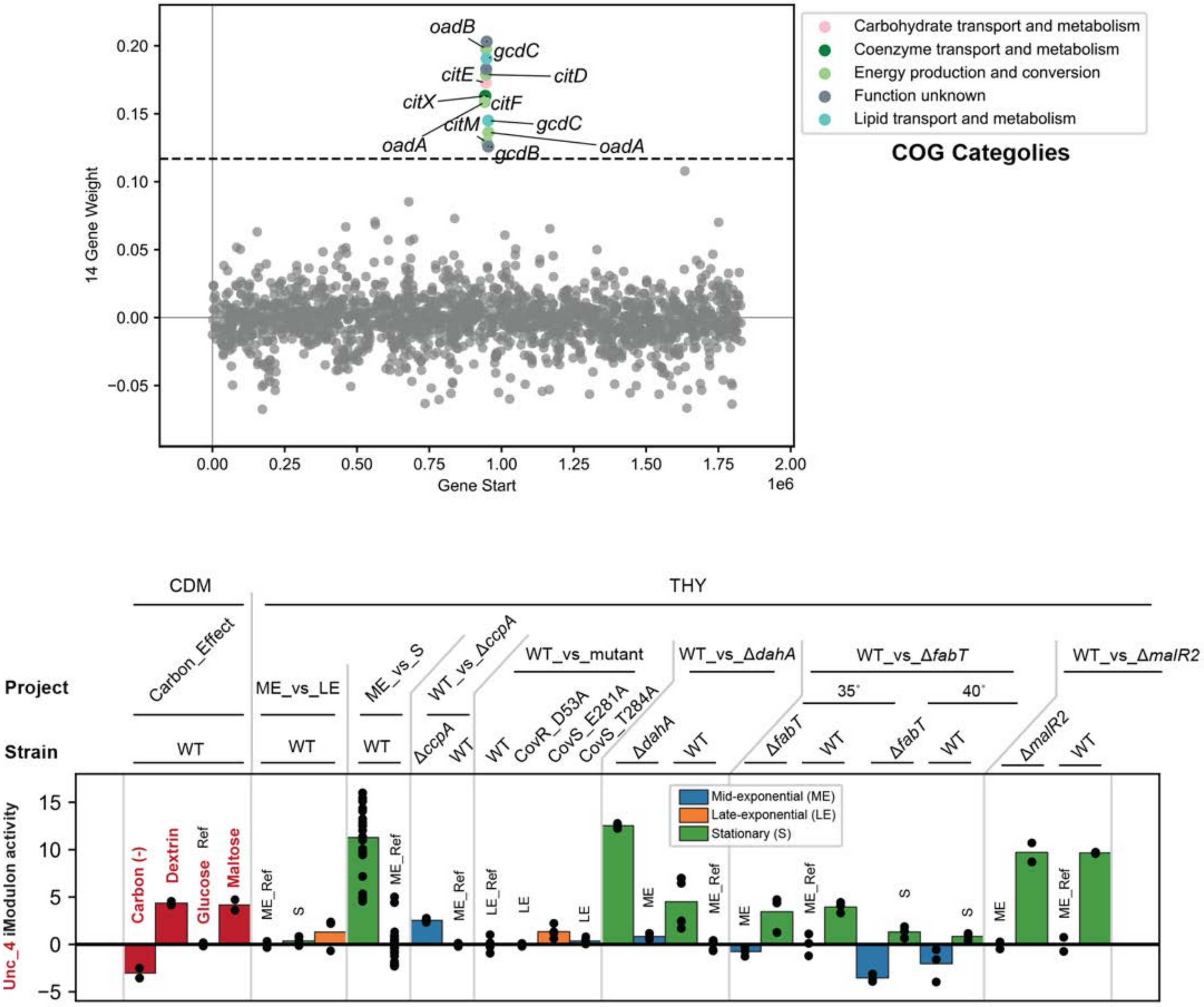

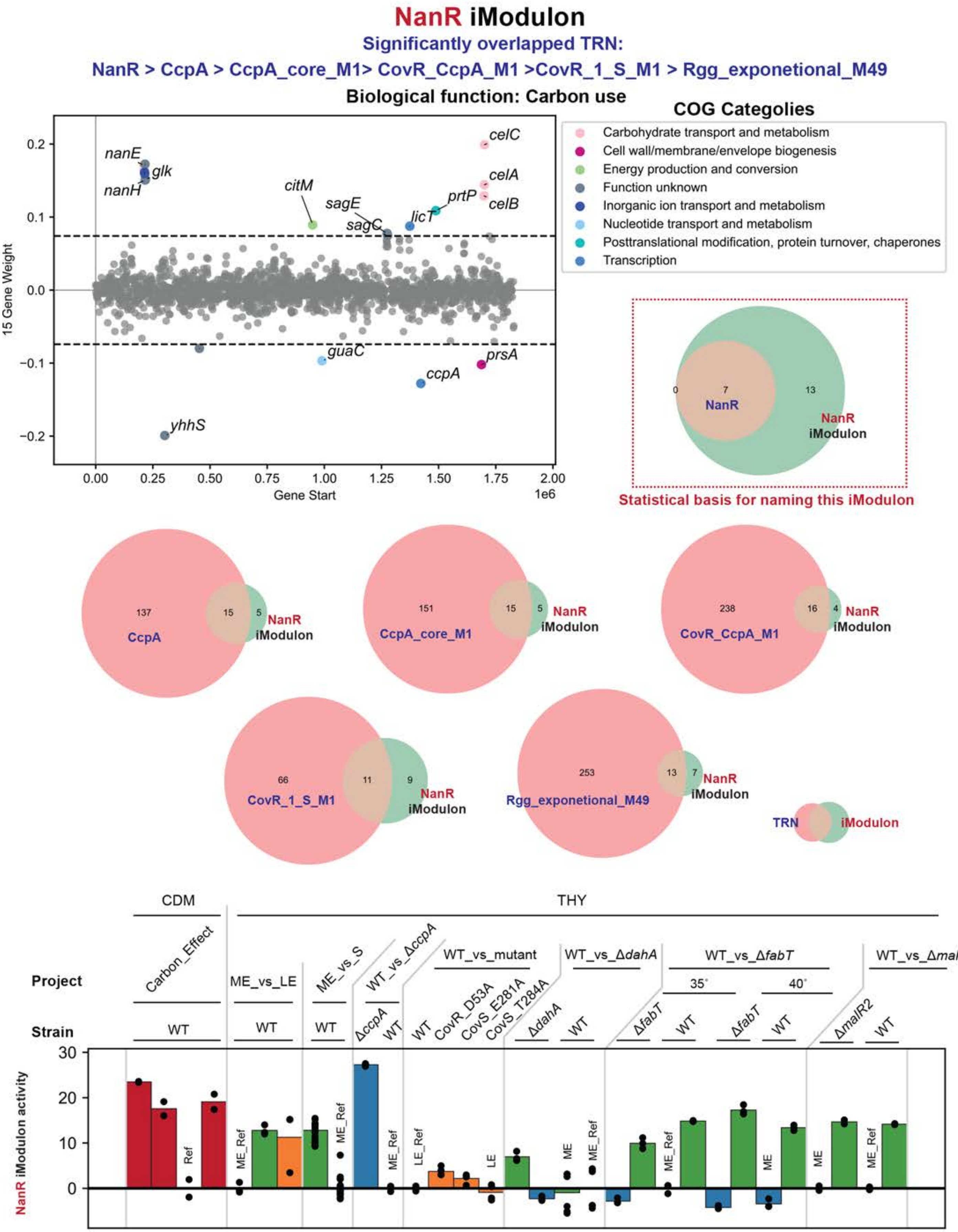
NanR iModulon
Significantly overlapped TRN:
NanR > CcpA > CcpA_core_M1> CovR_CcpA_M1 >CovR_1_S_M1 > Rgg_exponetional_M49
Biological function: Carbon use
COG Categolies
Carbohydrate transport and metabolism
Cell wall/membrane/envelope biogenesis
Energy production and conversion
Function unknown
Inorganic ion transport and metabolism
Nucleotide transport and metabolism
Posttranslational modification, protein turnover, chaperones
Transcription
15 Gene Weight
Gene Start
1e6
nanE
glk
nanH
citM
sagE
sagC
licT
prtP
celC
celA
celB
guaC
prsA
ccpA
yhhS
NanR
NanR iModulon
Statistical basis for naming this iModulon
CcpA
CcpA_core_M1
CovR_CcpA_M1
CovR_1_S_M1
Rgg_exponetional_M49
TRN
iModulon
CDM
THY
Project
Strain
Carbon_Effect
ME_vs_LE
ME_vs_S
WT_vs_ΔccpA
WT_vs_mutant
WT_vs_ΔdahA
WT_vs_ΔfabT
35°
40°
WT_vs_ΔmalR2
WT
ΔccpA
CovR_D53A
CovS_E281A
CovS_T284A
ΔdahA
ΔfabT
ΔmalR2
NanR iModulon activity
Ref
ME_Ref
LE_Ref
ME
LE

NrdR iModulon

Significantly overlapped TRN: NrdR

Biological function: Carbon use

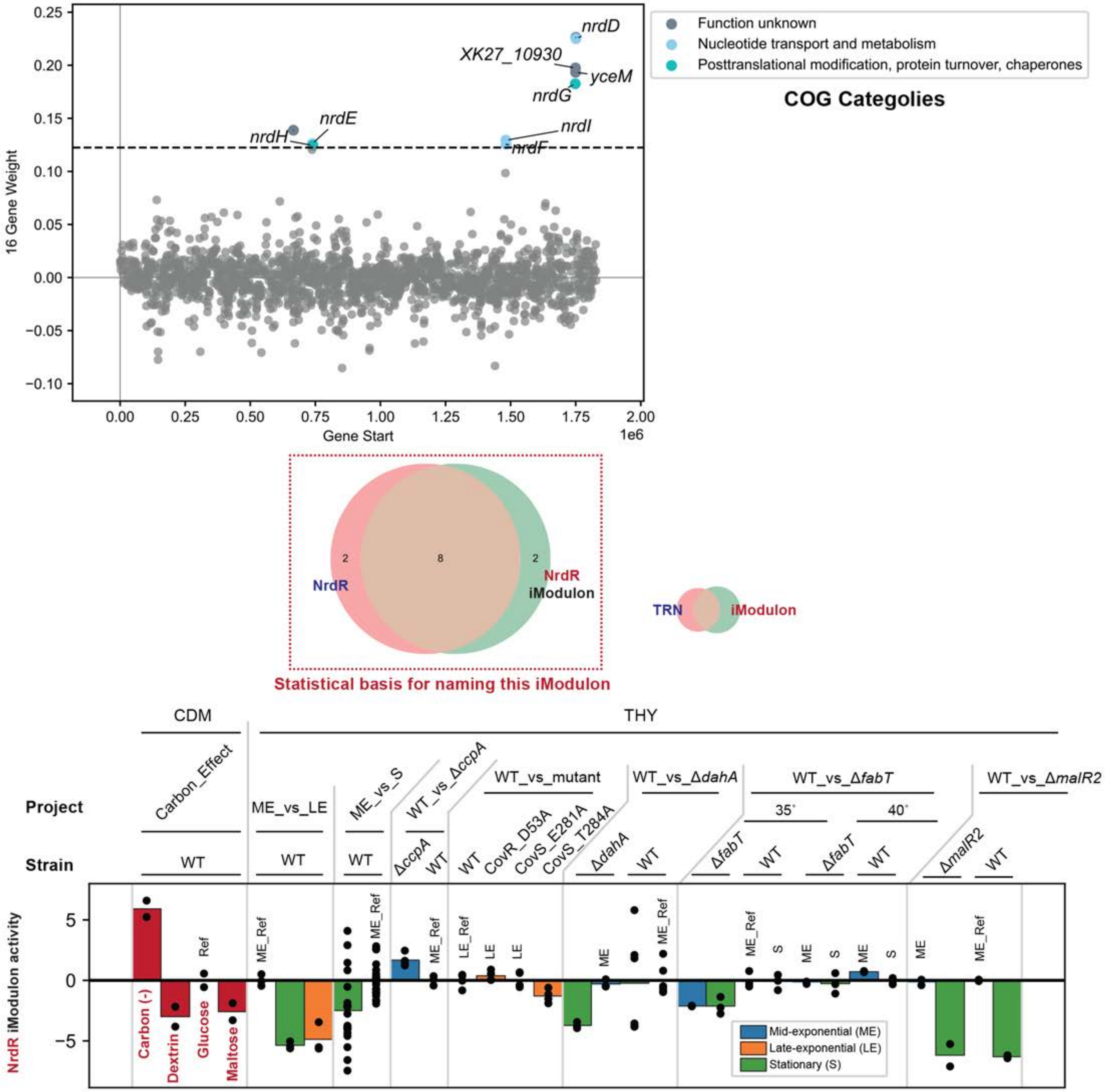

Unc_5 iModulon

Significantly overlapped TRN: ---

Biological function: ---

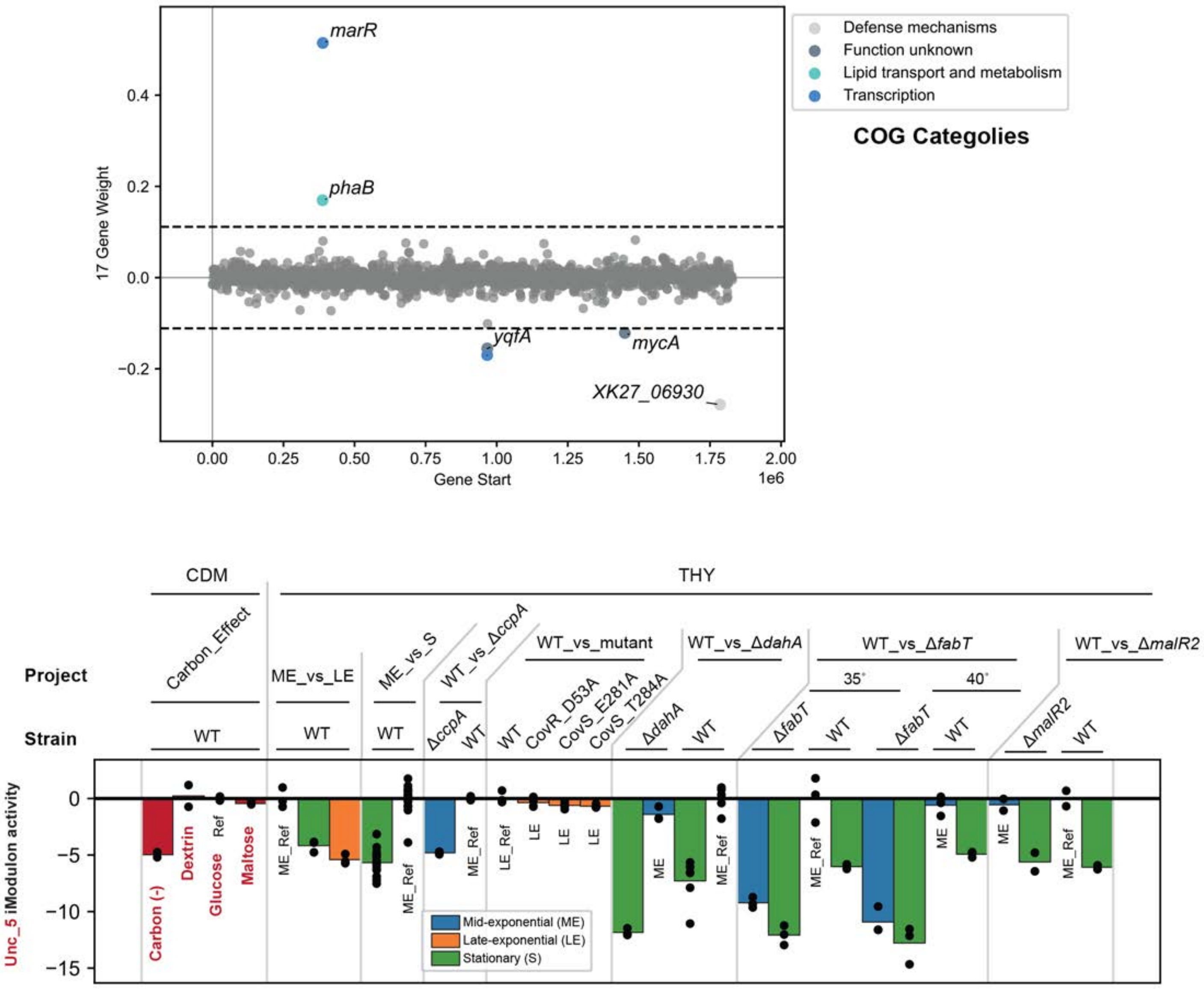

PurR iModulon

Significantly overlapped TRN: PurR

Biological function: Nucleotides

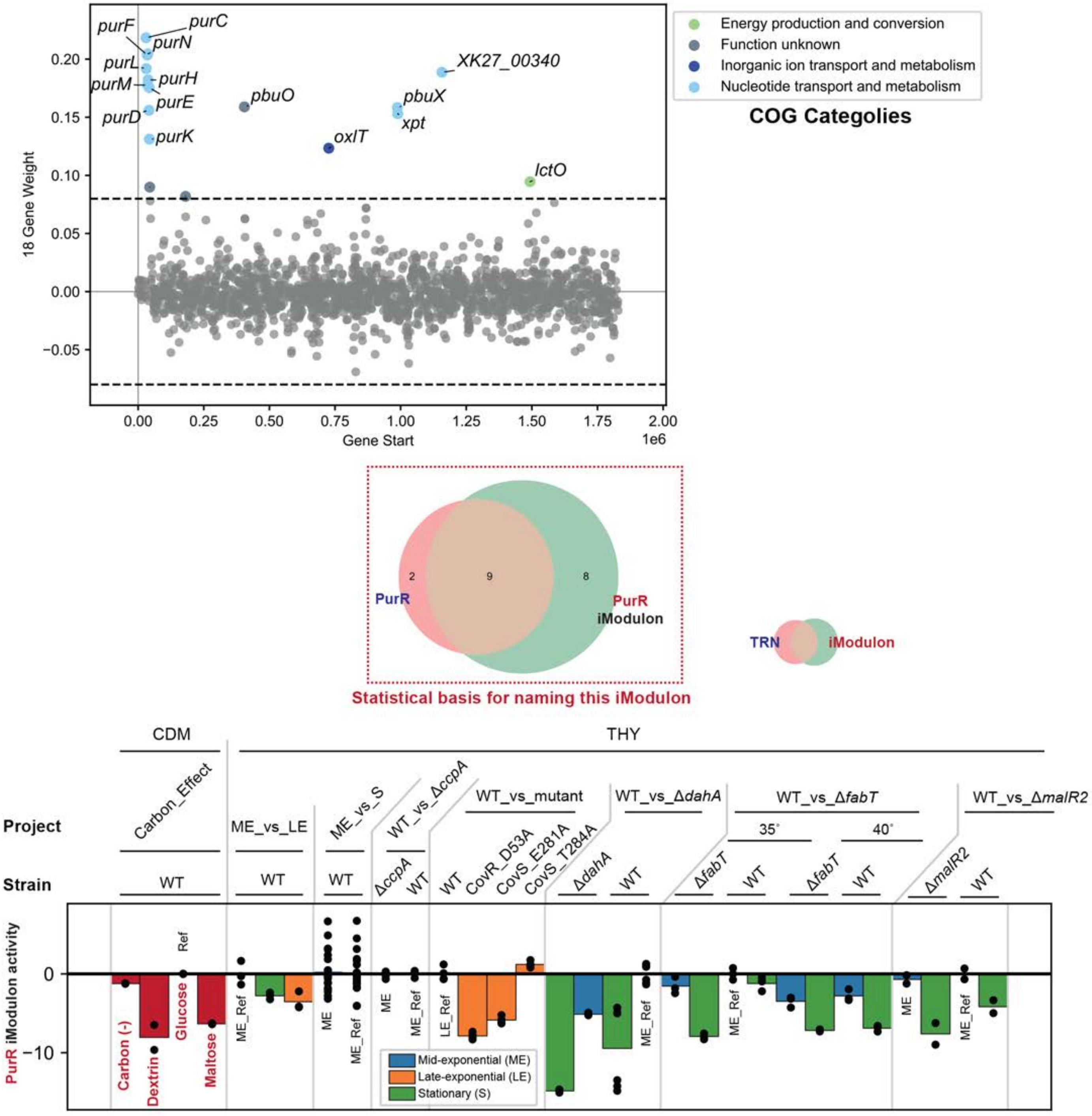

Unc_6 iModulon

Significantly overlapped TRN: ---

Biological function: General stress

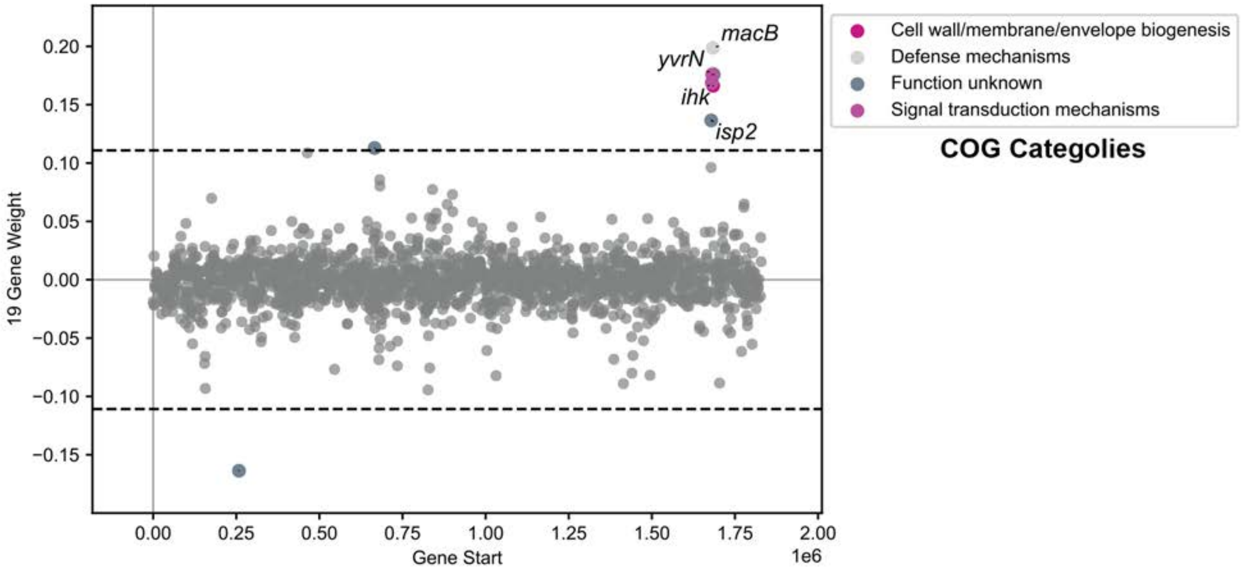

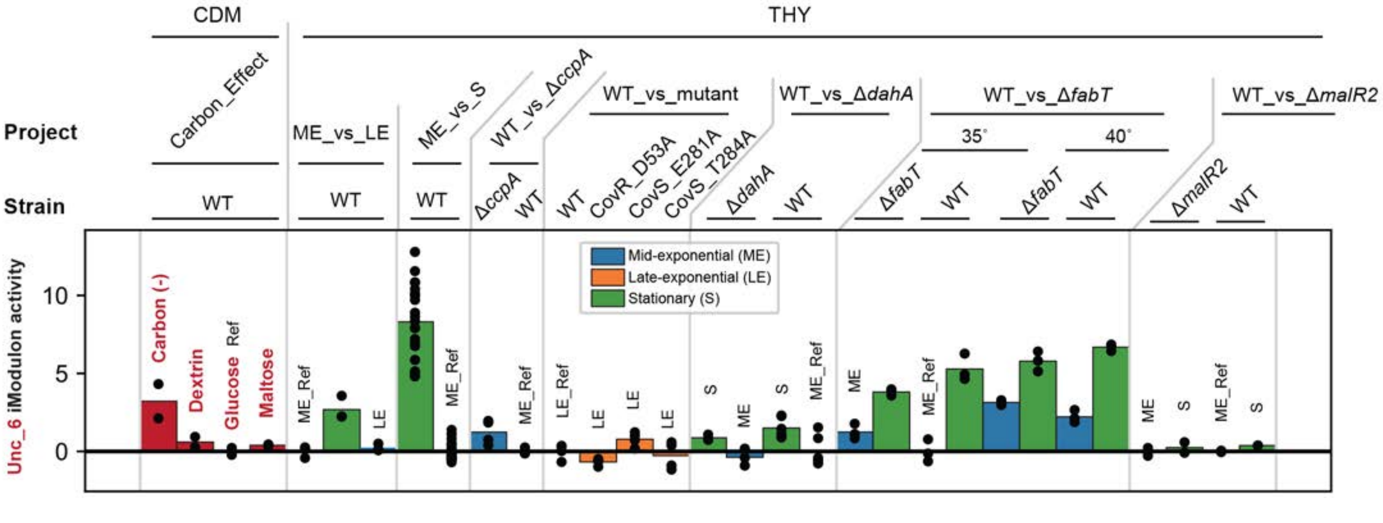

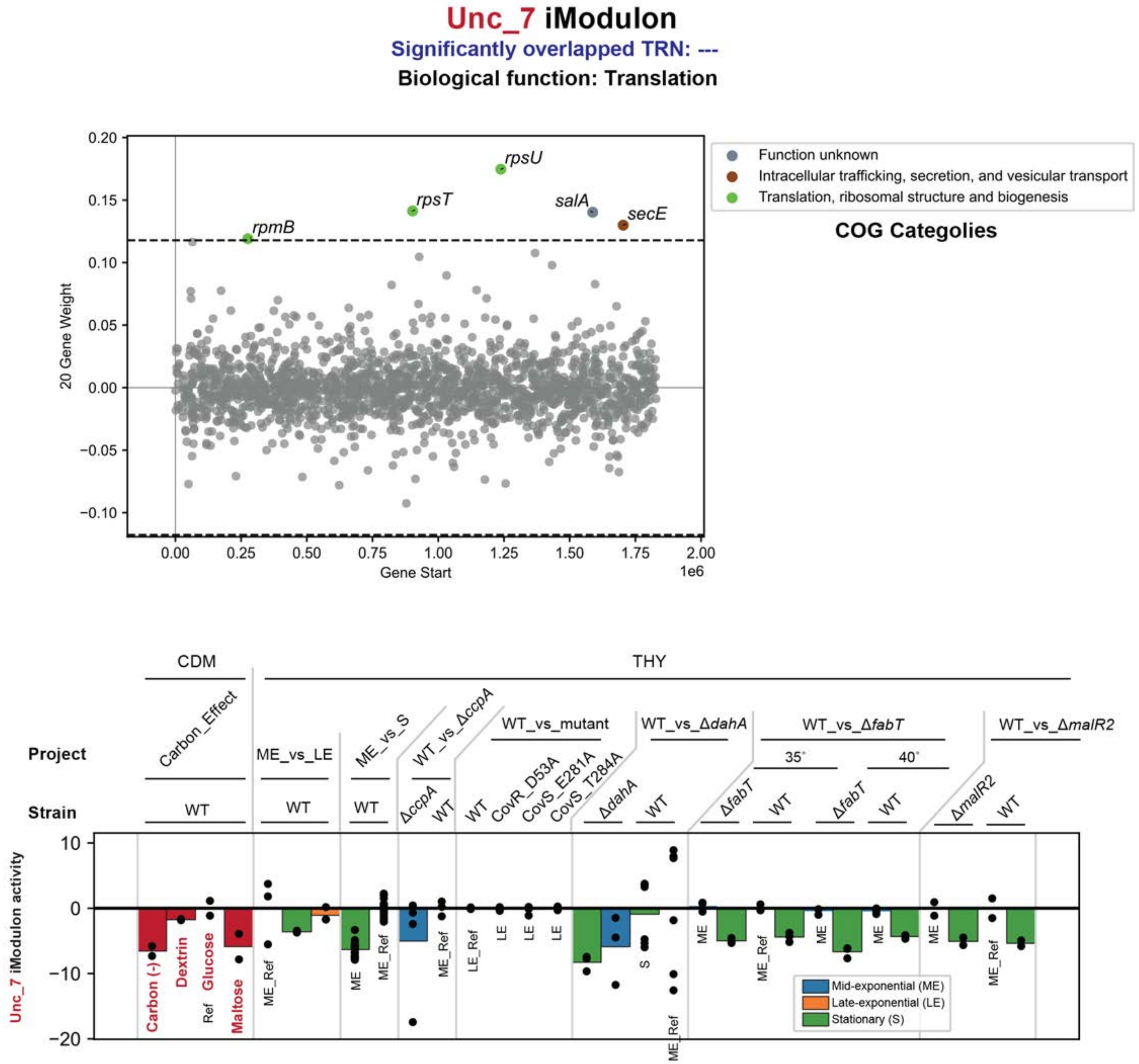
Unc_7 iModulon
Significantly overlapped TRN: ---
Biological function: Translation
20 Gene Weight
Gene Start
1e6
rpsU
rpsT
rpmB
salA
secE
Function unknown
Intracellular trafficking, secretion, and vesicular transport
Translation, ribosomal structure and biogenesis
COG Categolies
CDM
THY
Project
Strain
Carbon_Effect
ME_vs_LE
ME_vs_S
WT_vs_ΔccpA
WT_vs_mutant
WT_vs_ΔdahA
WT_vs_ΔfabT
WT_vs_ΔmalR2
35°
40°
WT
ΔccpA
CovR_D53A
CovS_E281A
CovS_T284A
ΔdahA
ΔfabT
ΔmalR2
Unc_7 iModulon activity
Carbon (-)
Dextrin
Glucose
Ref
Maltose
ME_Ref
ME
LE_Ref
LE
S
Mid-exponential (ME)
Late-exponential (LE)
Stationary (S)

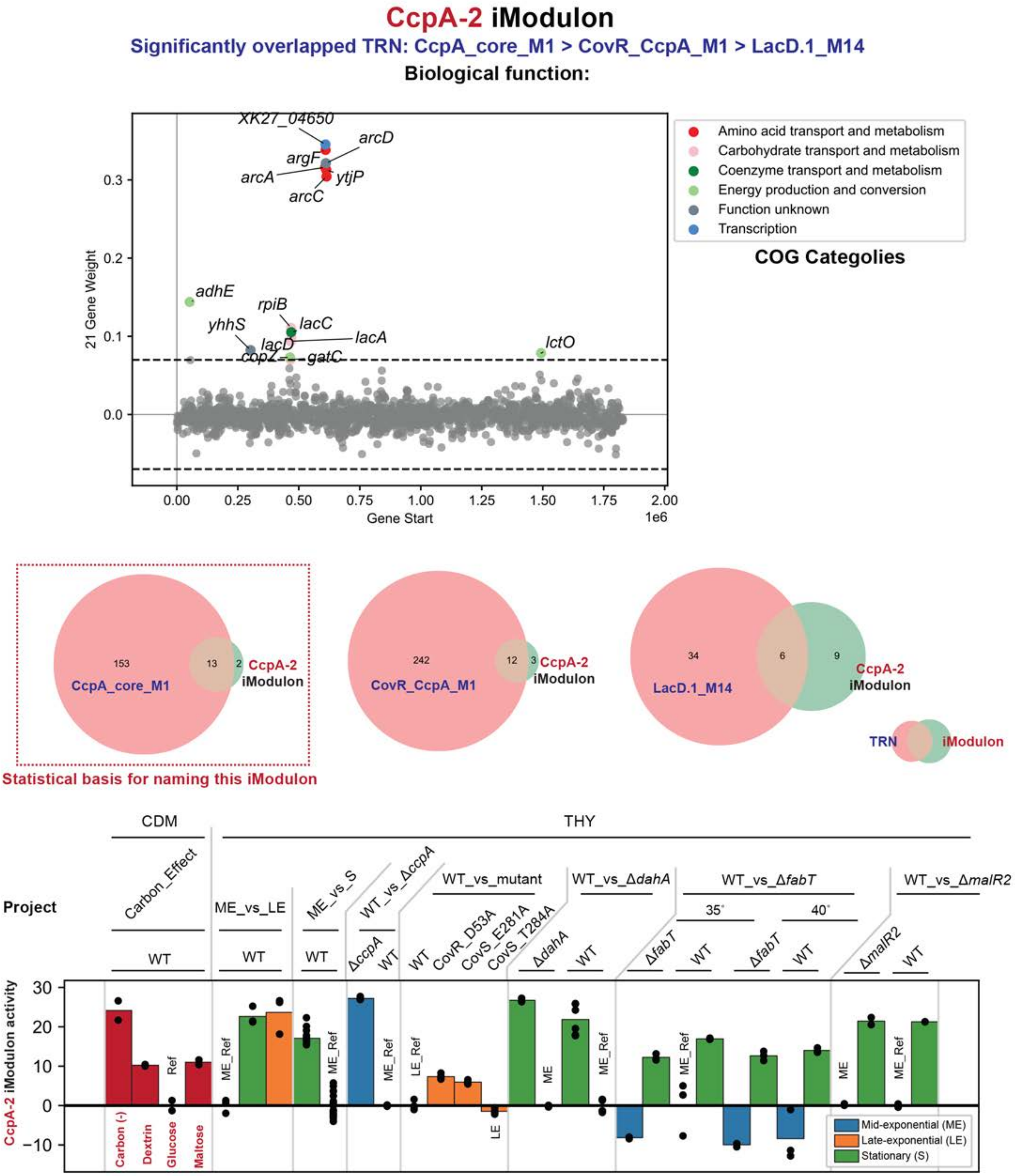
CcpA-2 iModulon
Significantly overlapped TRN: CcpA_core_M1 > CovR_CcpA_M1 > LacD.1_M14
Biological function:
XK27_04650
arcD
argF
arcA
ytjP
arcC
adhE
rpiB
lacC
yhhS
lacA
lacD
copZ
gatC
lctO
21 Gene Weight
Gene Start
1e6
Amino acid transport and metabolism
Carbohydrate transport and metabolism
Coenzyme transport and metabolism
Energy production and conversion
Function unknown
Transcription
COG Categolies
153
13
2
CcpA-2
iModulon
CcpA_core_M1
242
12
3
CovR_CcpA_M1
34
6
9
LacD.1_M14
TRN
Statistical basis for naming this iModulon
CDM
THY
Project
Carbon_Effect
ME_vs_LE
ME_vs_S
WT_vs_ΔccpA
WT_vs_mutant
WT_vs_ΔdahA
WT_vs_ΔfabT
35°
40°
WT_vs_ΔmalR2
WT
ΔccpA
CovR_D53A
CovS_E281A
CovS_T284A
ΔdahA
ΔfabT
ΔmalR2
CcpA-2 iModulon activity
Ref
ME_Ref
LE_Ref
ME
LE
Carbon (-)
Dextrin
Glucose
Maltose
Mid-exponential (ME)
Late-exponential (LE)
Stationary (S)

Phage_2-2 iModulon

Significantly overlapped TRN: Phage_2

Biological function: Prophages

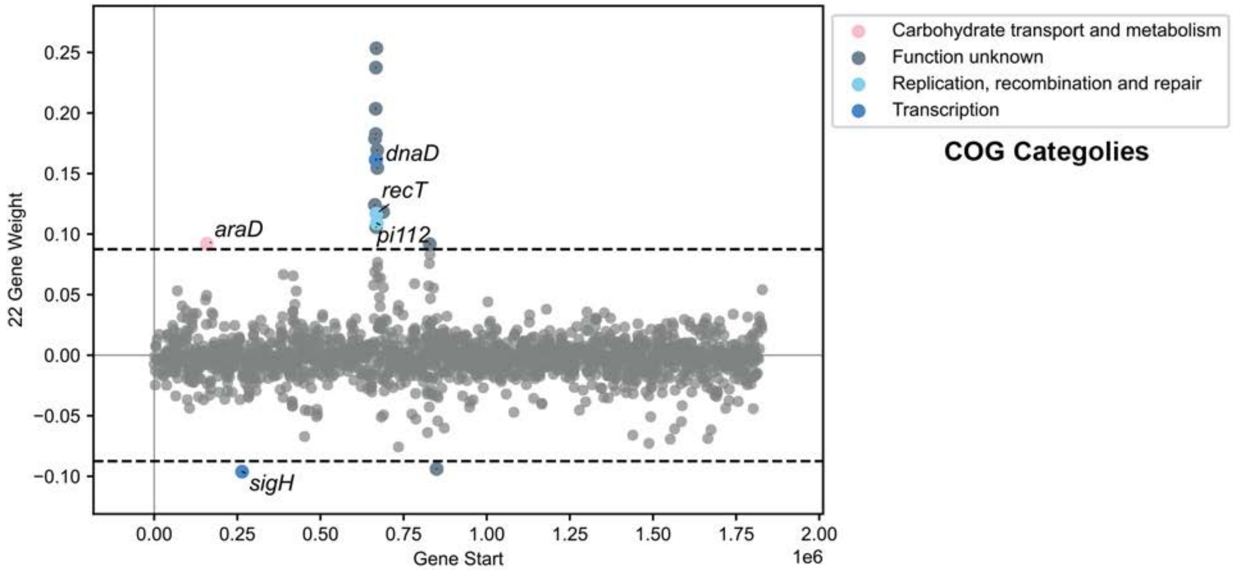

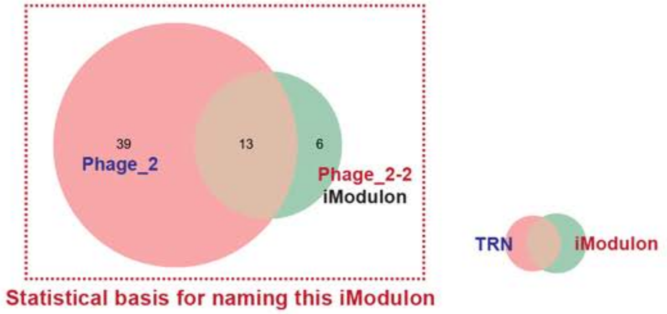

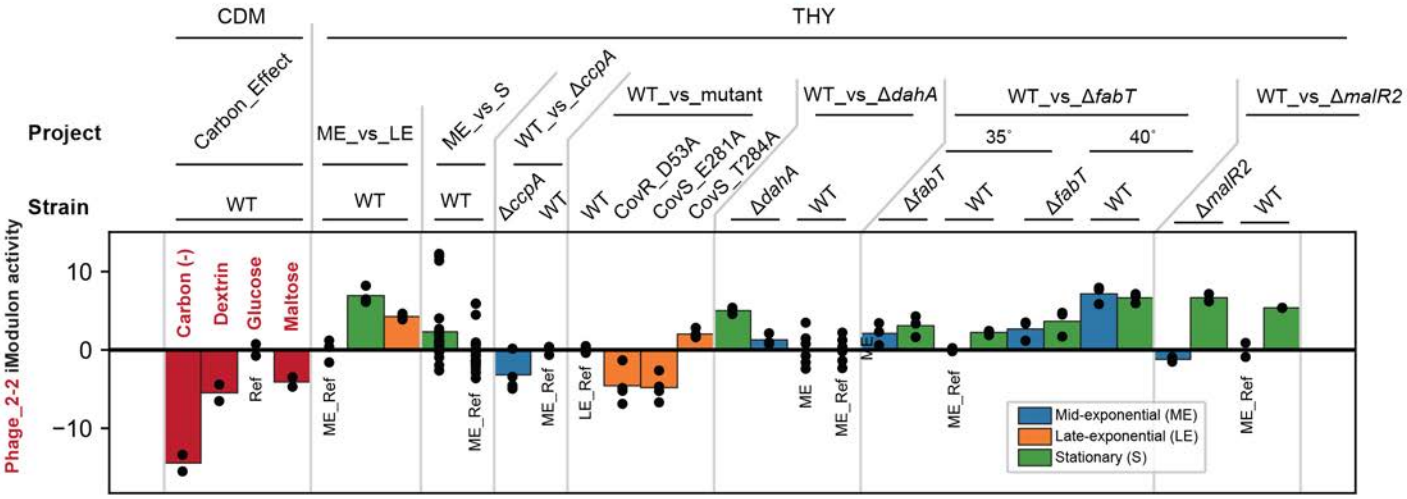

SLS iModulon

Significantly overlapped TRN:
CovR_1_S_M1 > CovR_1_ME_M1 > CovR_CcpA_M1 > CcpA_core_M1 > Rgg_exponetional_M49 > CovR_2_LE_M1 > CcpA > Mga_low_Glucose_M1 > CovR_2_S_M1 > Rgg_postexponential_M49

Biological function: Toxin production

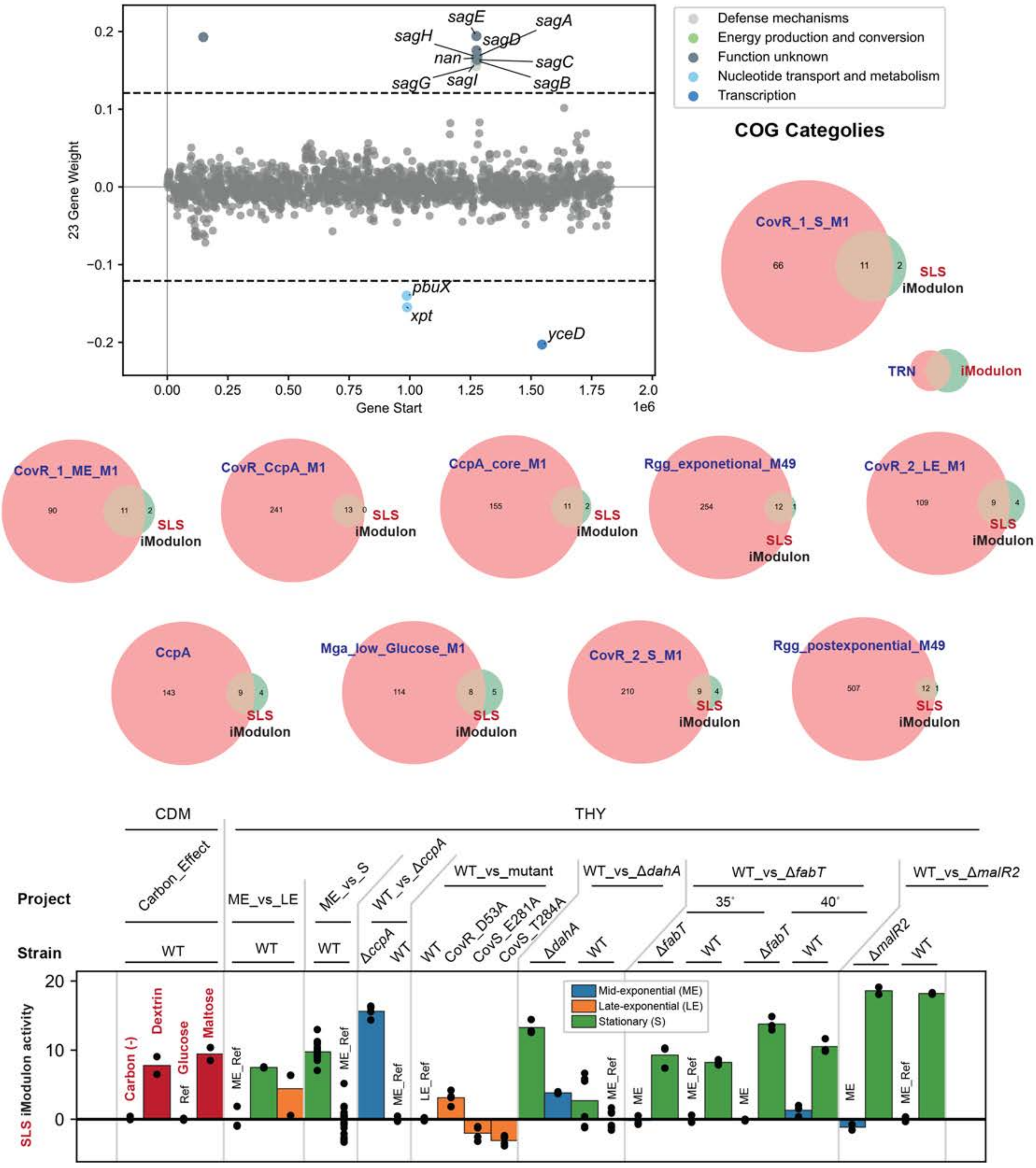

Unc_8 iModulon

Significantly overlapped TRN: ---

Biological function: Translation

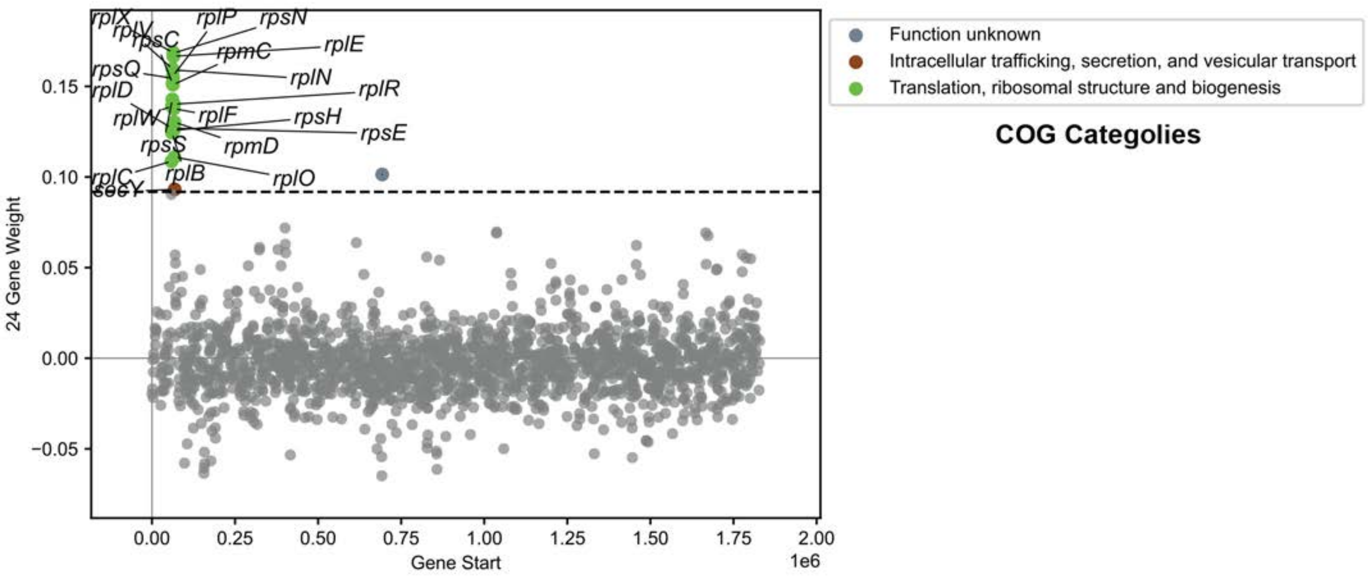

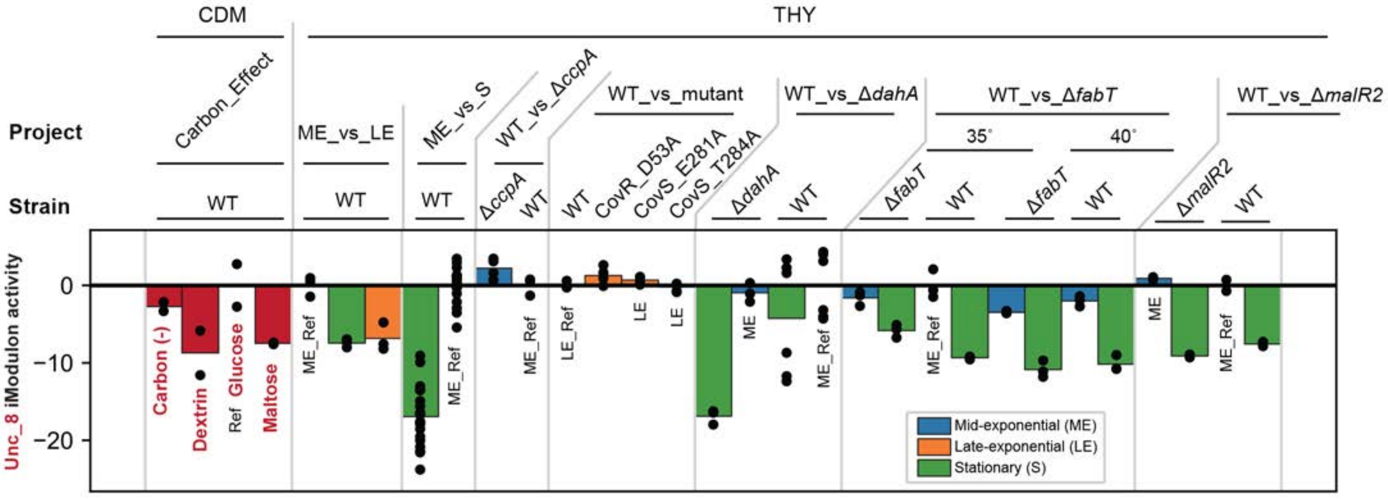

Unc_9 iModulon

Significantly overlapped TRN:

Biological function:

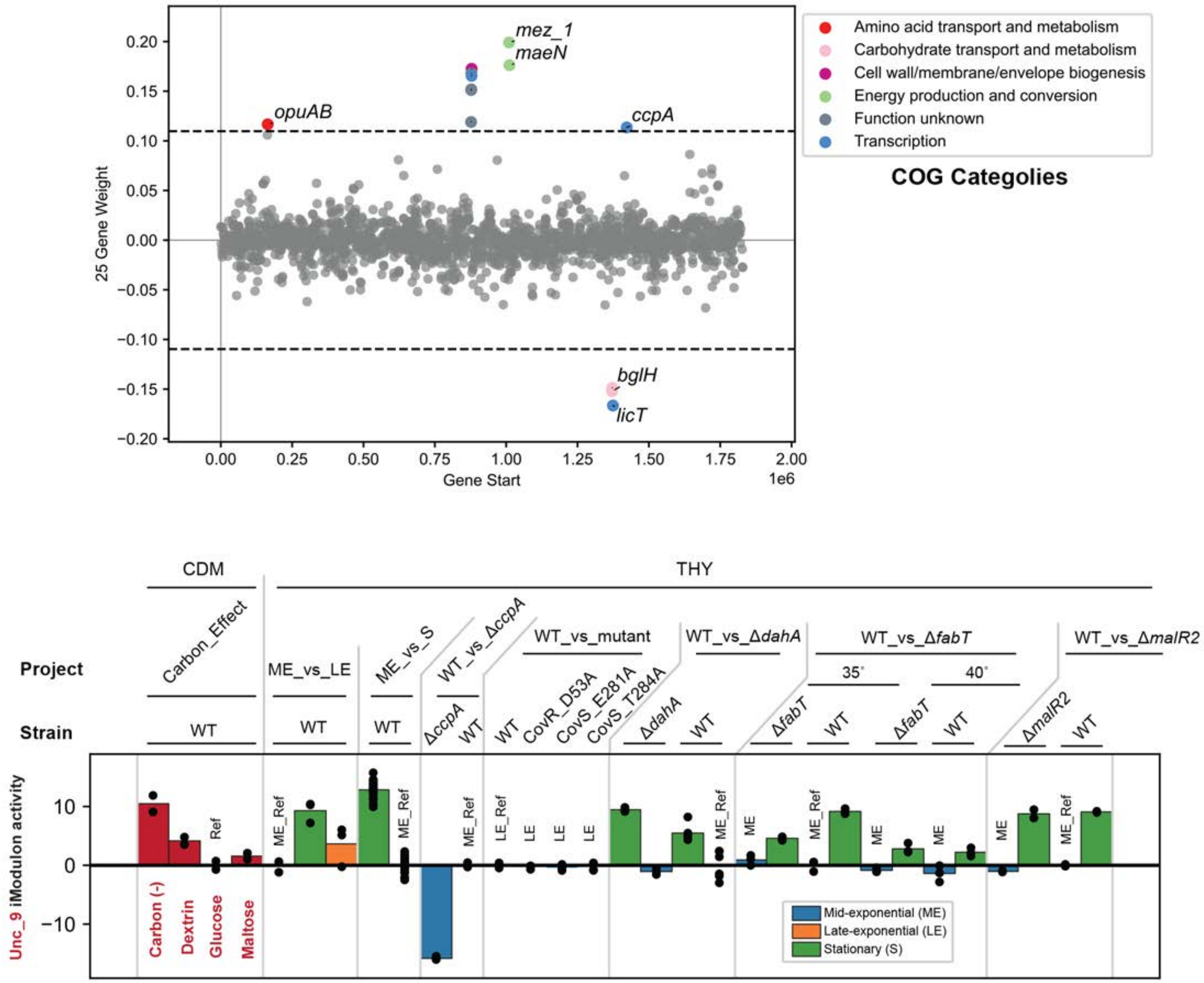

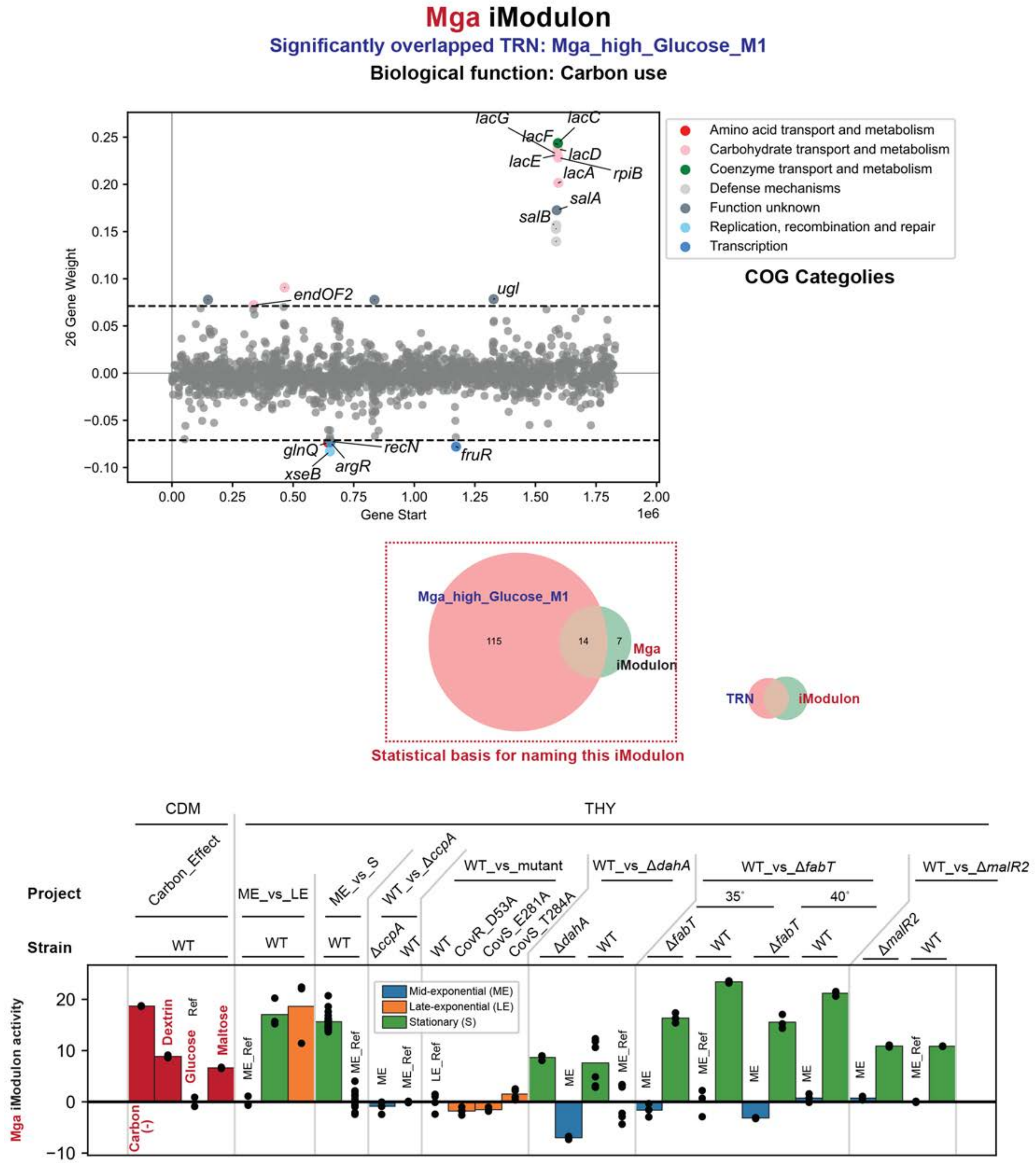

Mga iModulon
Significantly overlapped TRN: Mga_high_Glucose_M1
Biological function: Carbon use
26 Gene Weight
Gene Start
1e6
lacG
lacC
lacF
lacD
lacE
lacA
rpiB
salA
salB
endOF2
ugl
glnQ
recN
fruR
xseB
argR
Amino acid transport and metabolism
Carbohydrate transport and metabolism
Coenzyme transport and metabolism
Defense mechanisms
Function unknown
Replication, recombination and repair
Transcription
COG Categolies
Mga_high_Glucose_M1
115
14
7
Mga
iModulon
TRN
iModulon
Statistical basis for naming this iModulon
CDM
THY
Project
Strain
Carbon_Effect
ME_vs_LE
ME_vs_S
WT_vs_ΔccpA
WT_vs_mutant
WT_vs_ΔdahA
WT_vs_ΔfabT
WT_vs_ΔmalR2
35°
40°
WT
ΔccpA
CovR_D53A
CovS_E281A
CovS_T284A
ΔdahA
ΔfabT
ΔmalR2
Mga iModulon activity
Carbon (-)
Dextrin
Glucose
Ref
Maltose
ME_Ref
LE_Ref
ME
Mid-exponential (ME)
Late-exponential (LE)
Stationary (S)

Unc_10 iModulon

Significantly overlapped TRN: ---

Biological function: ---

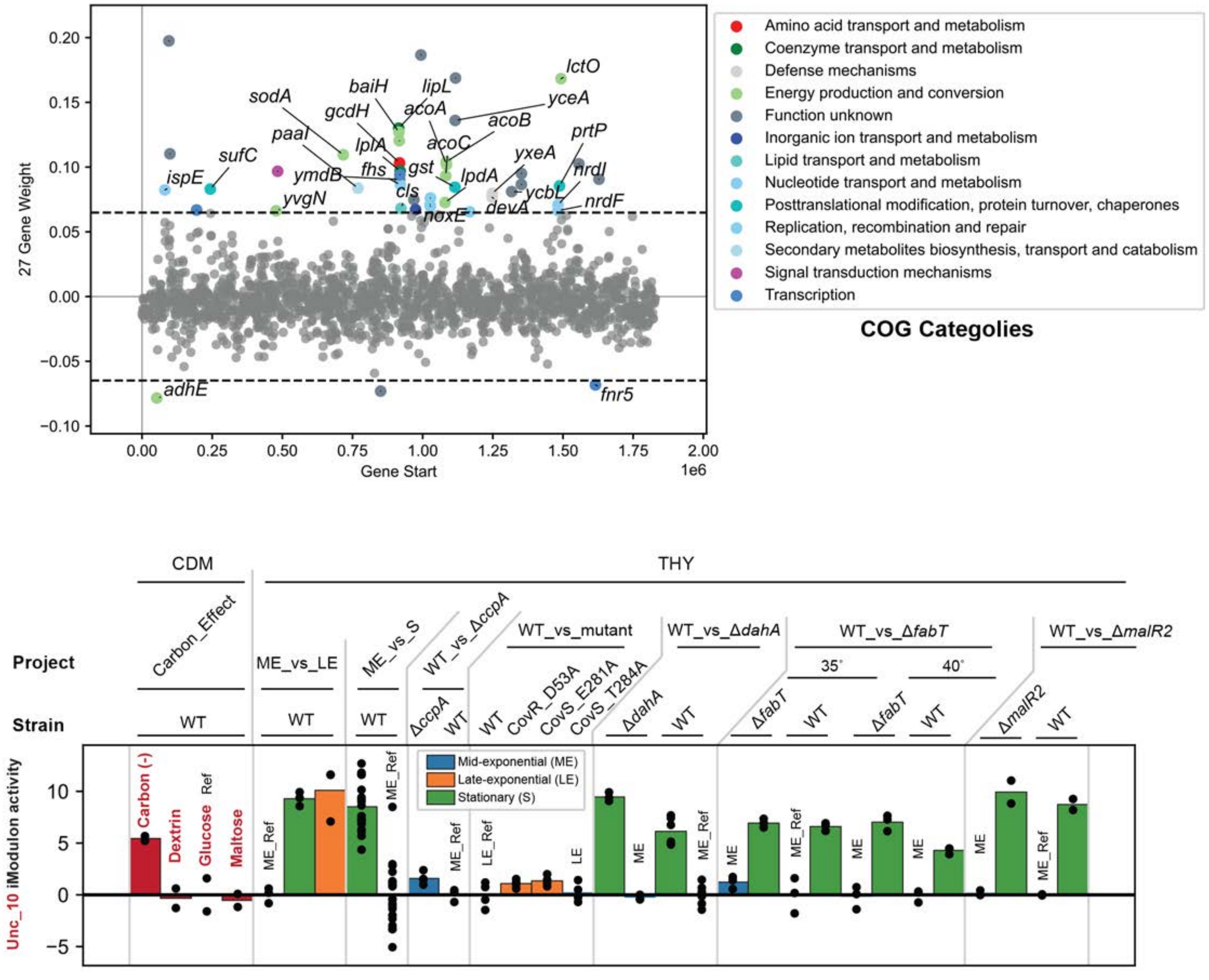

CovR-2 iModulon

Significantly overlapped TRN: CovR_1_ME_M1 > CovR_CcpA_M1 > CovR_1_S_M1 > Rgg_exponetional_M49

Biological function: Replication

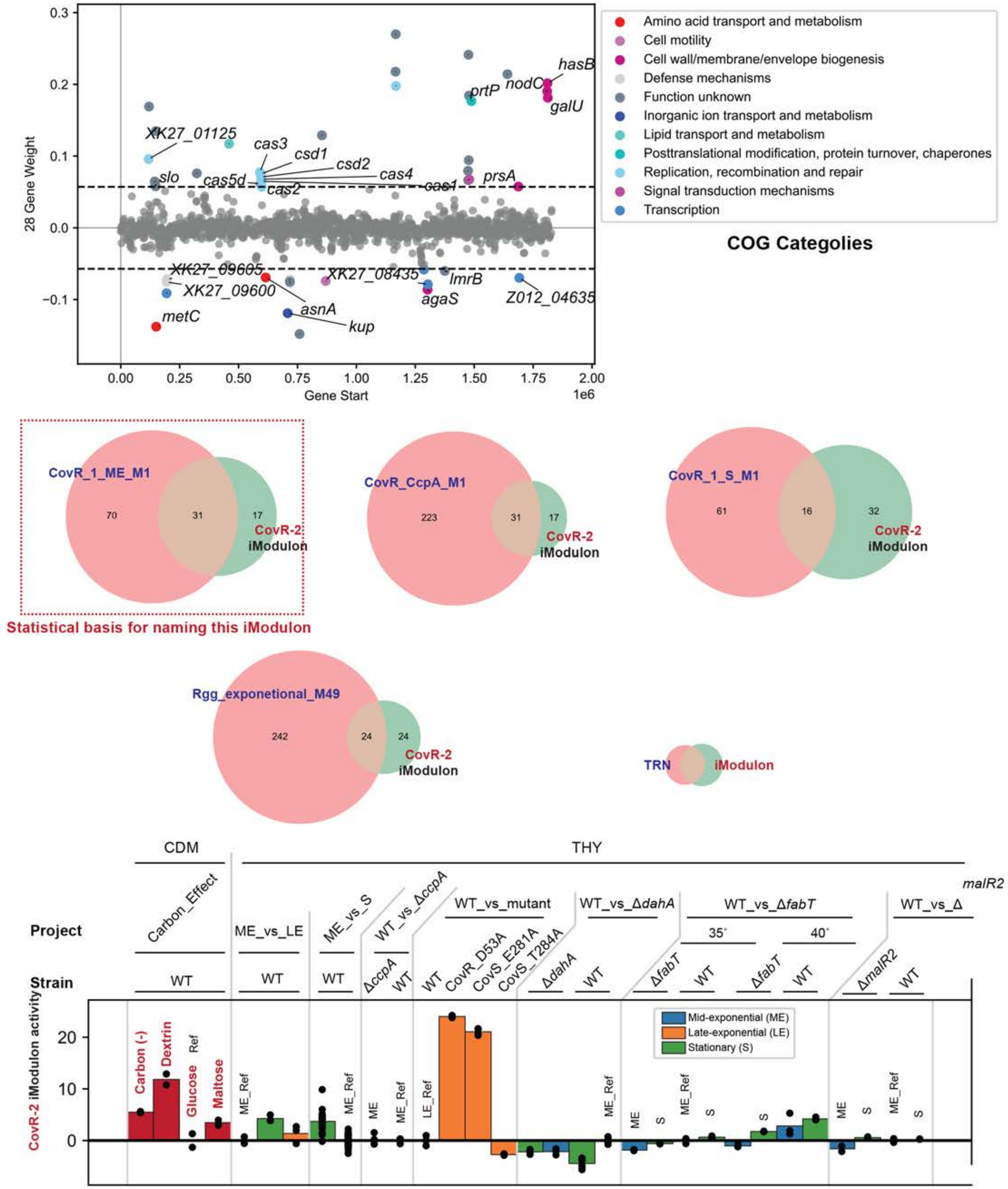

MalR2 iModulon

Significantly overlapped TRN: MalR2

Biological function: Carbon use

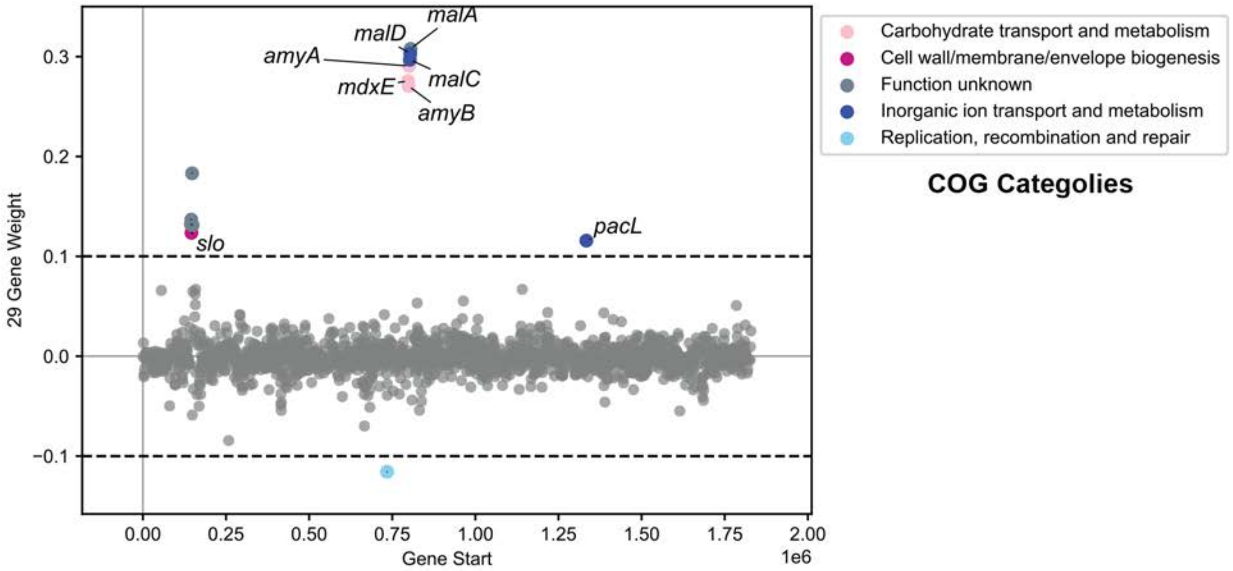

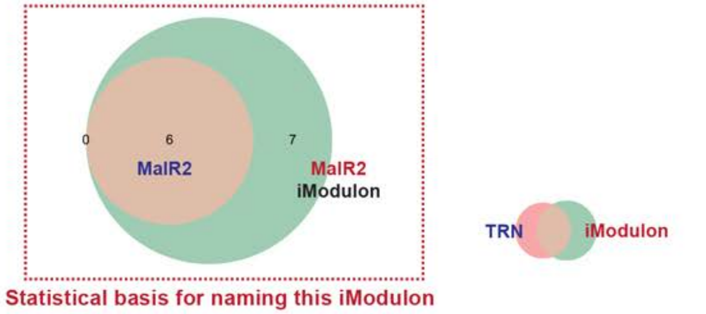

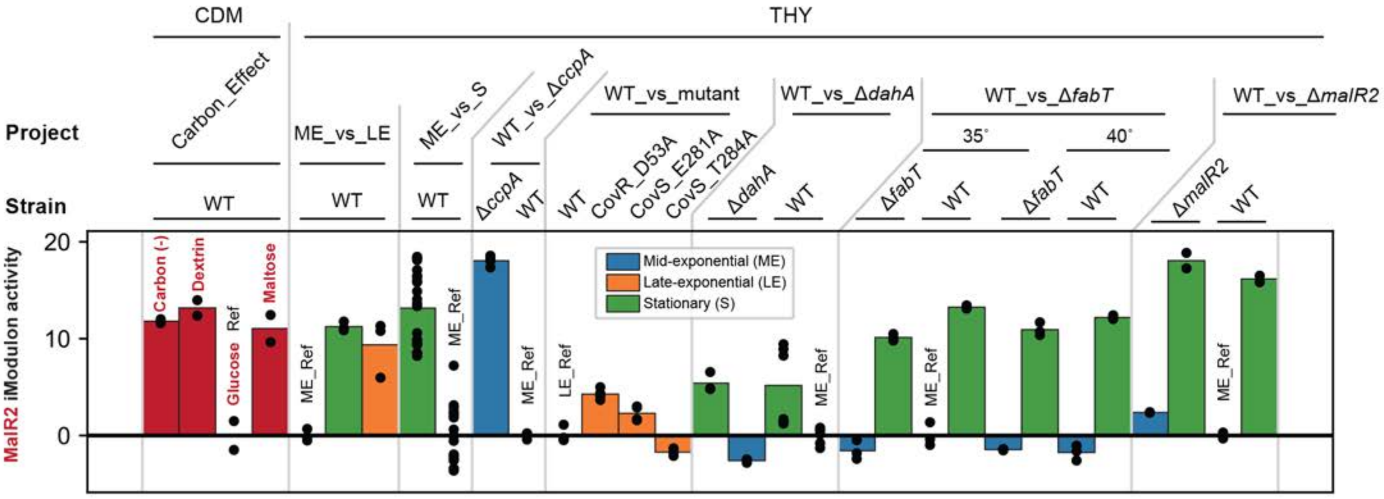

Phage_3-1 iModulon

Significantly overlapped TRN: Phage_3 > CovR_CcpA_M1

Biological function: Prophages

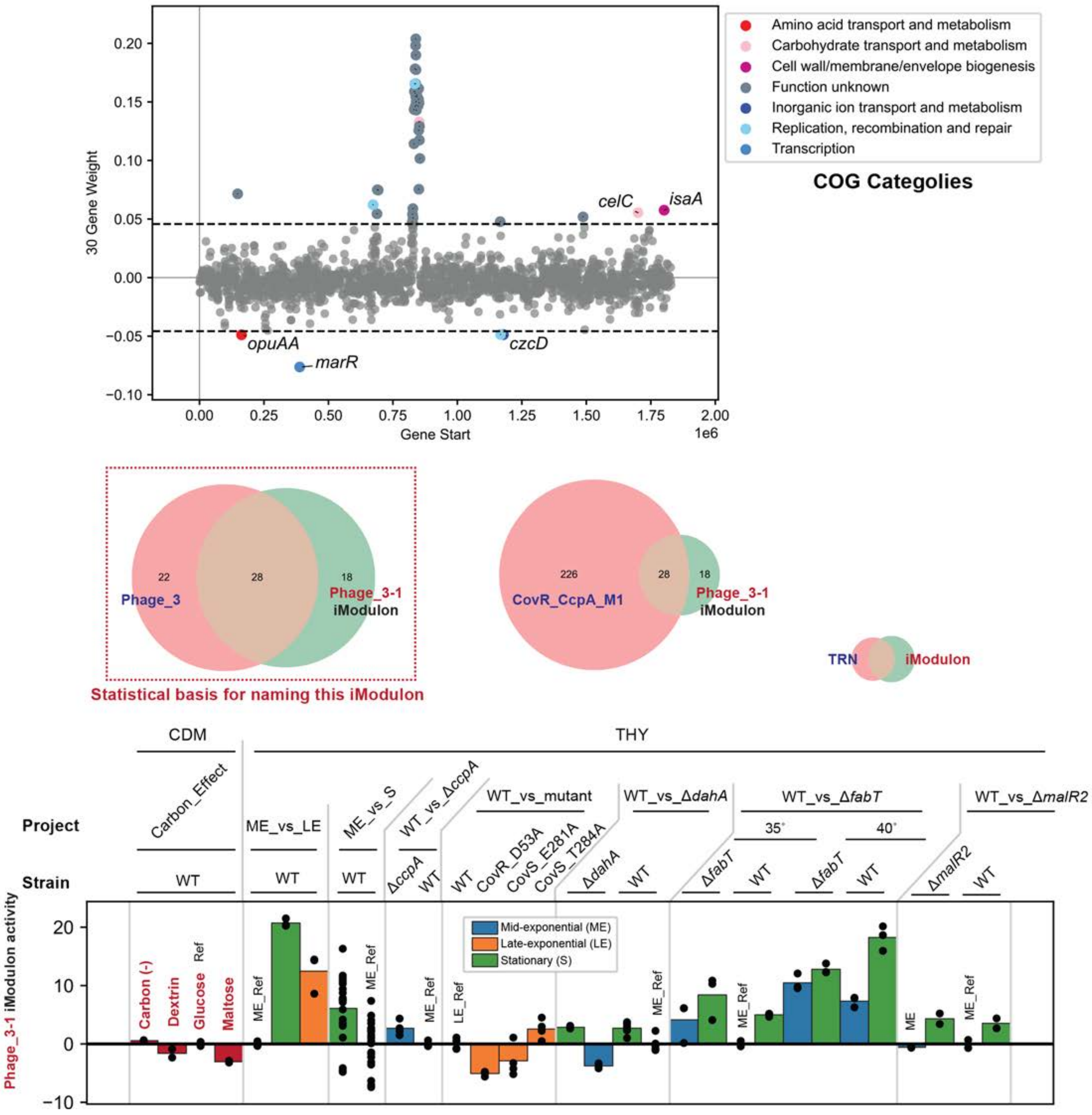

Phage_2-3 iModulon

Significantly overlapped TRN: Phage_2 > Phage_3

Biological function: Prophages

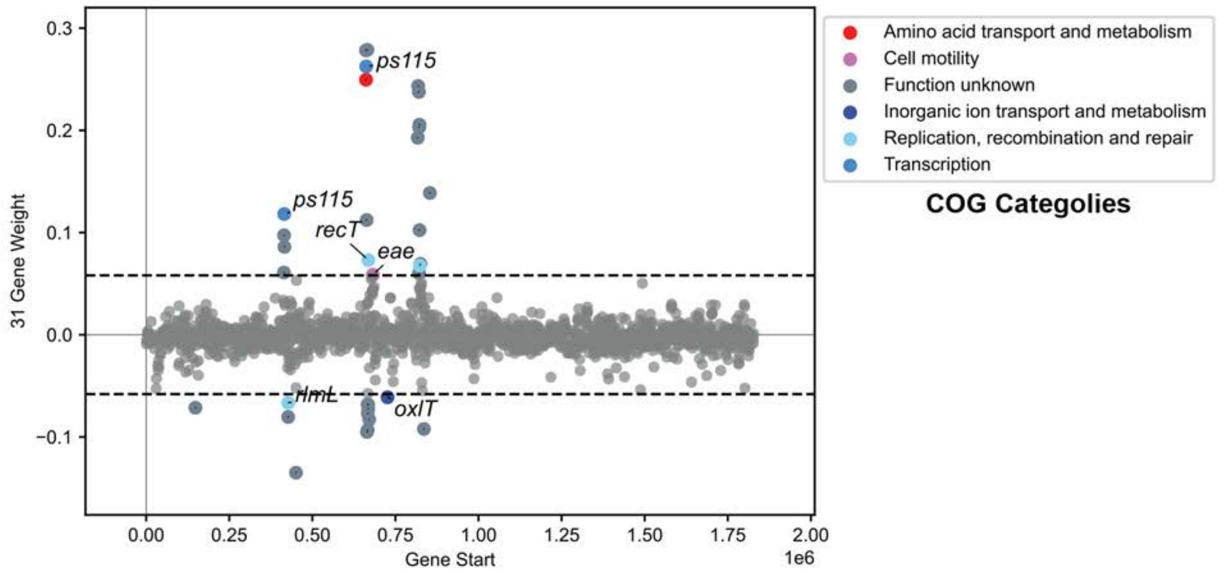

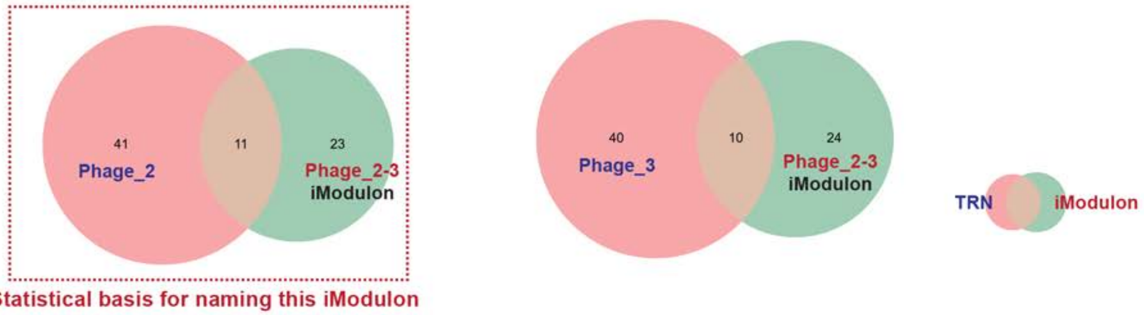

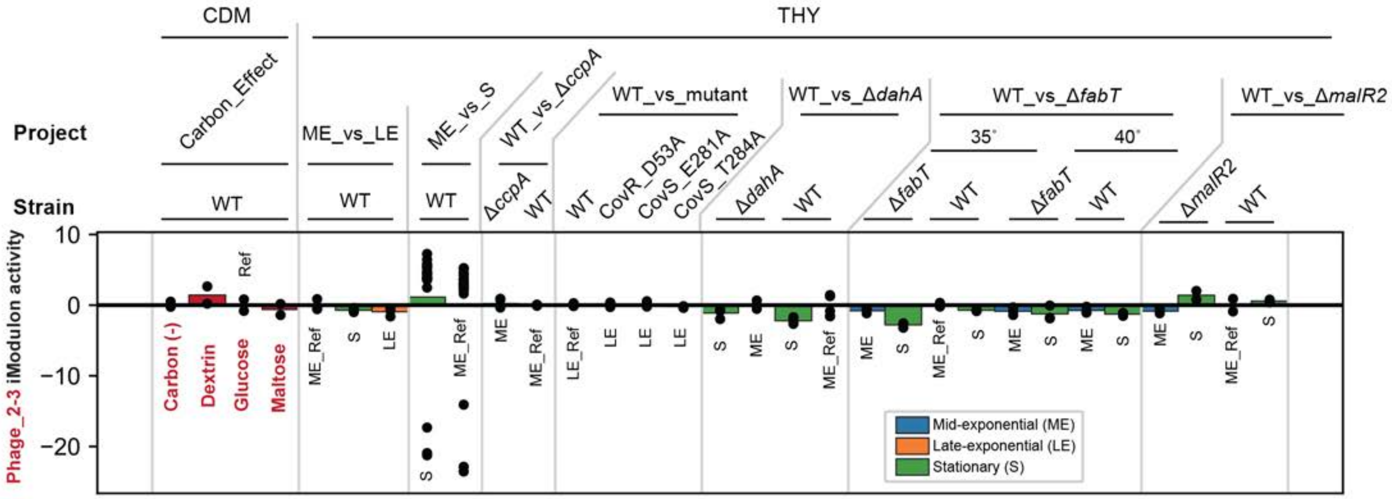

Unc_11 iModulon

Significantly overlapped TRN: ---

Biological function: Translation

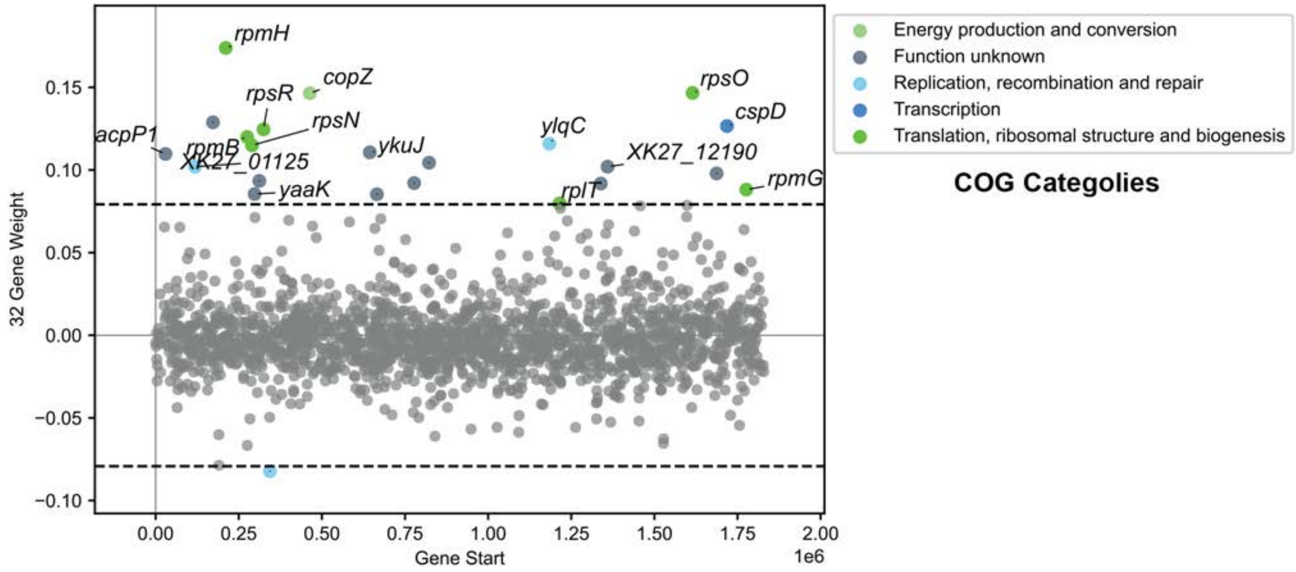

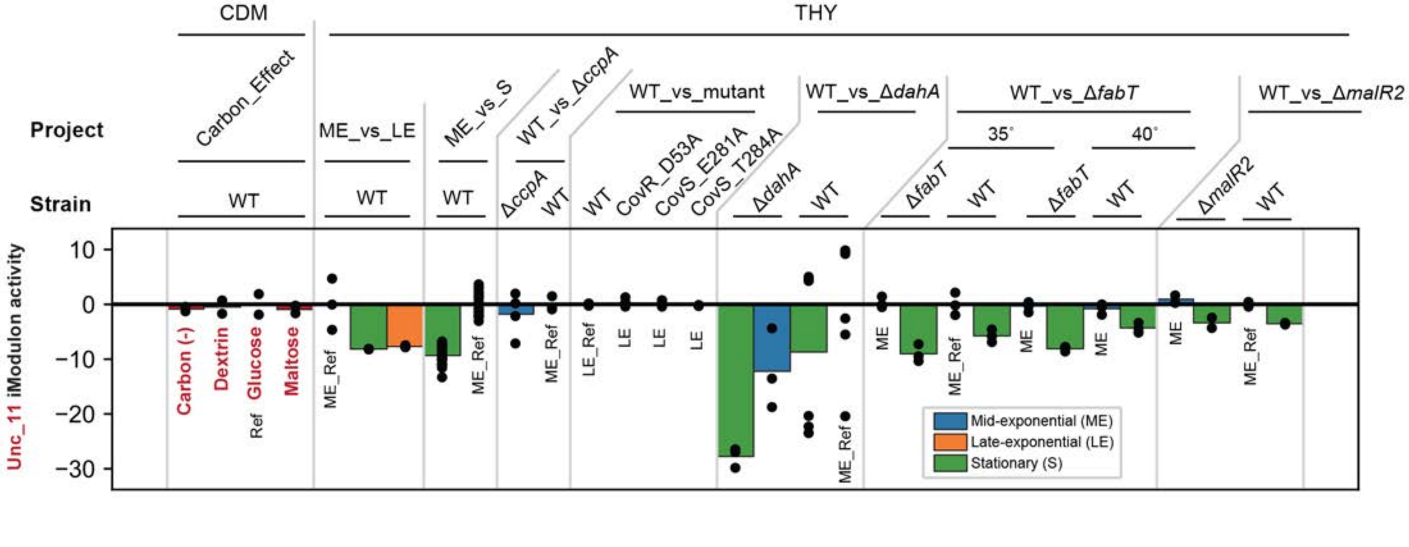

Galactose iModulon

Significantly overlapped TRN: ---

Biological function: Carbon use

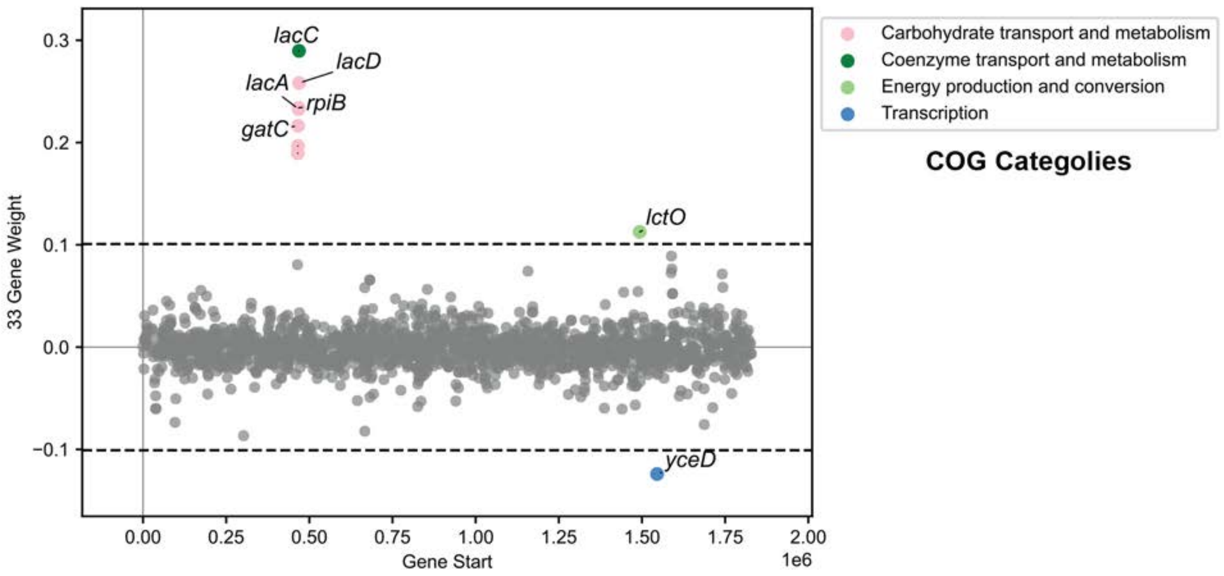

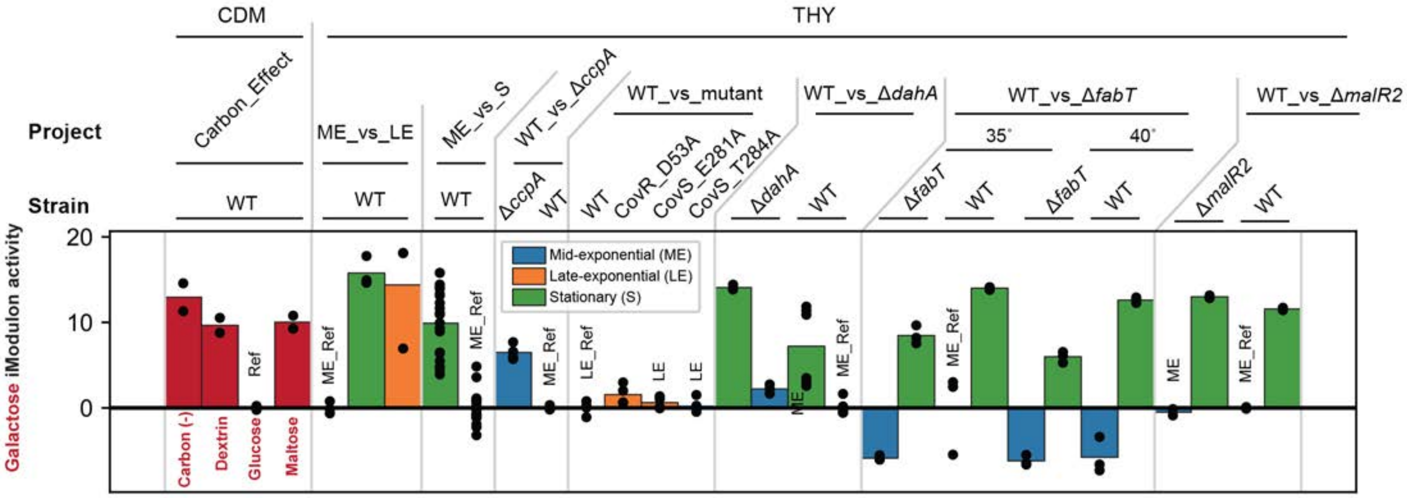

Transport-1 iModulon

Significantly overlapped TRN: ---

Biological function: Multi-purpose metabolism

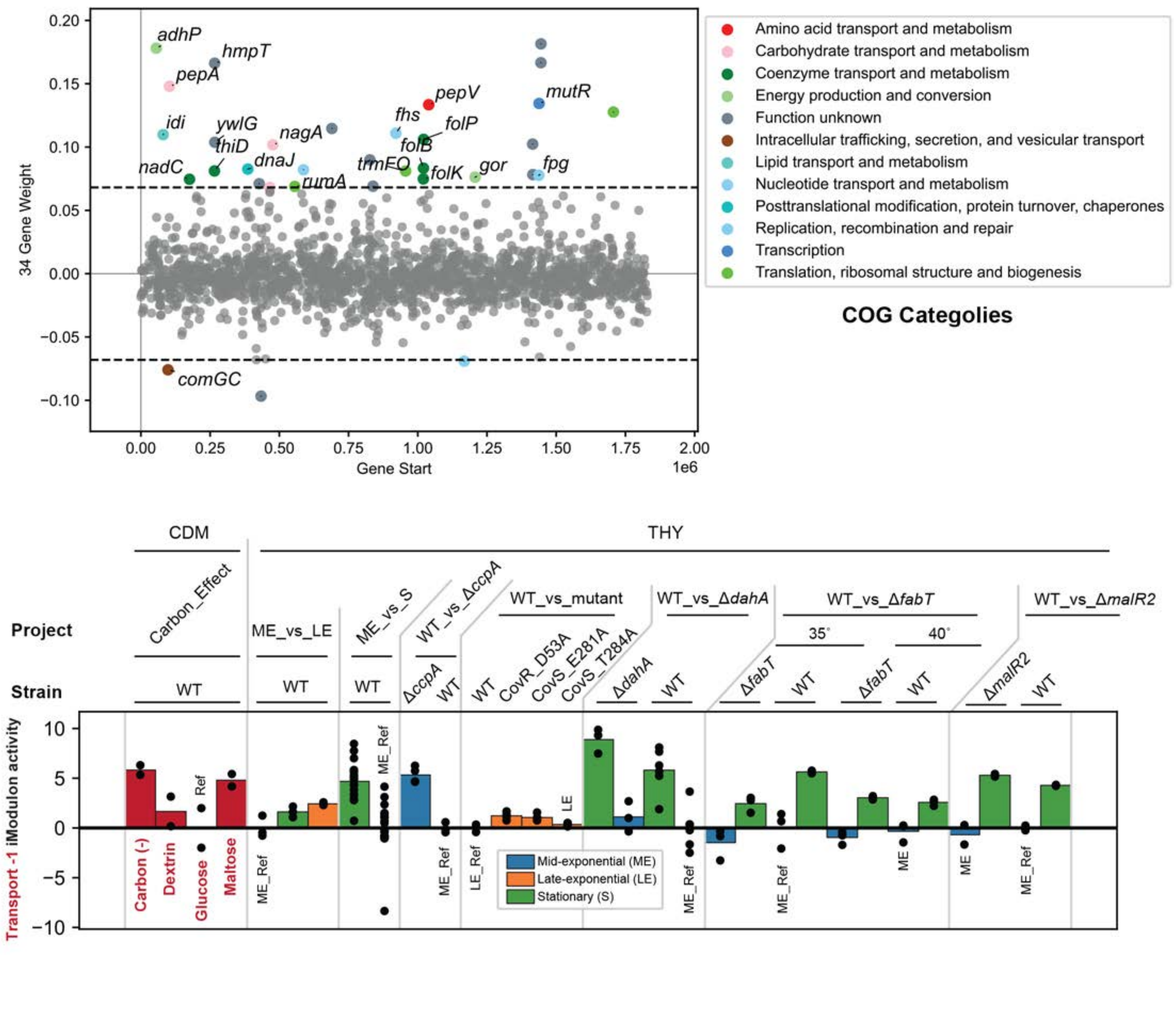

YwzG iModulon

Significantly overlapped TRN: YwzG

Biological function: Transport

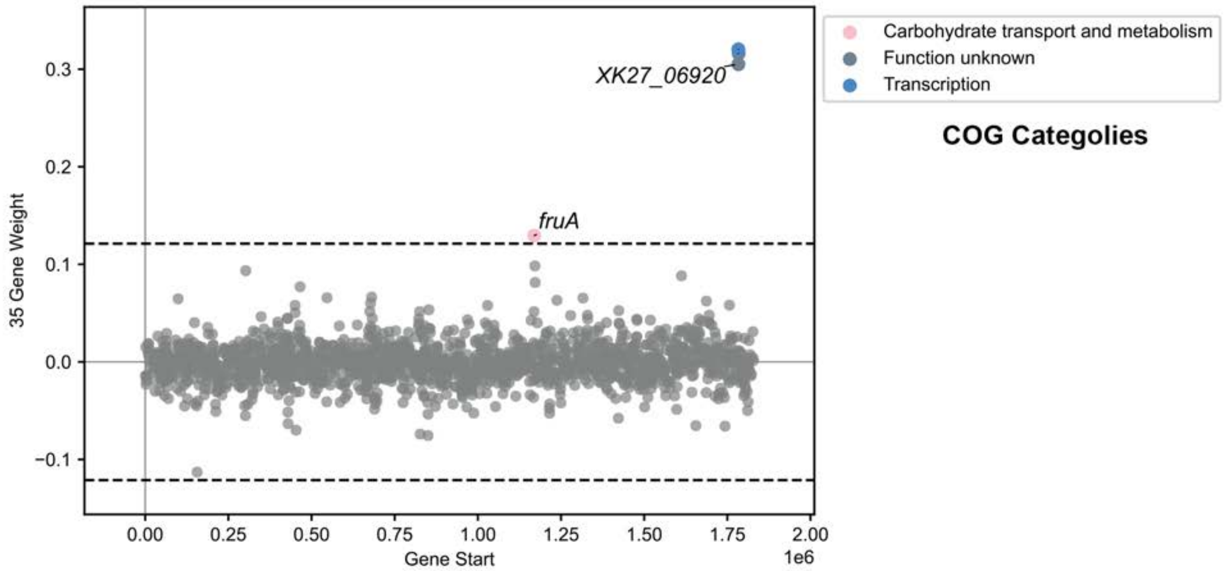

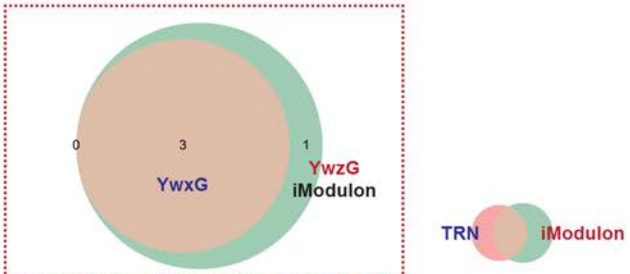

Statistical basis for naming this iModulon

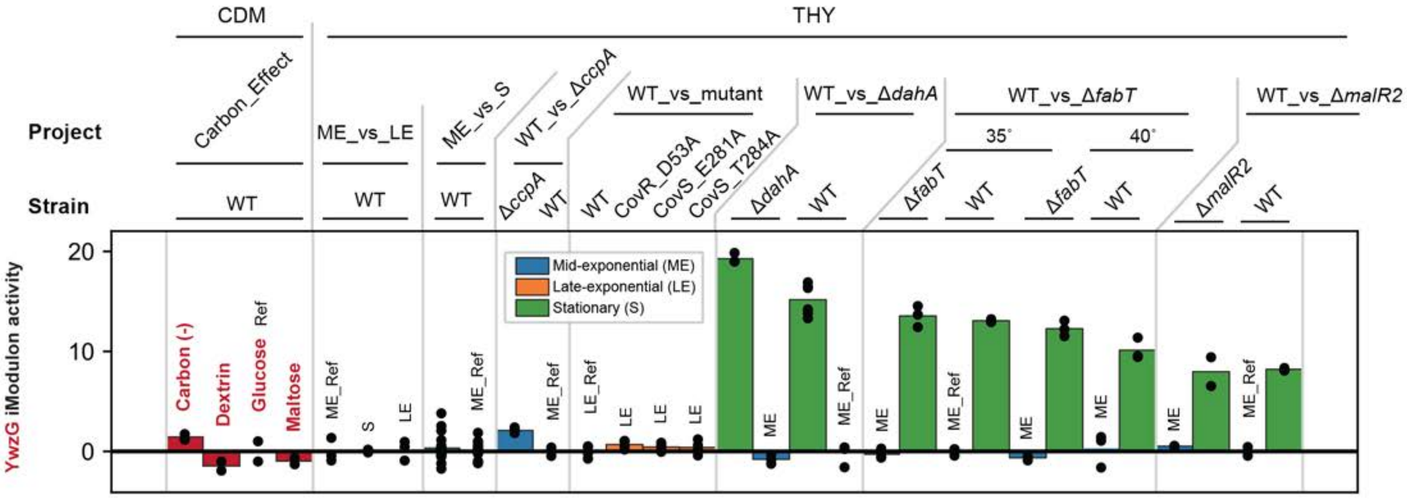

HrcA iModulon

Significantly overlapped TRN: HrcA > CtsR > Nra_transition_M49

Biological function: Translation

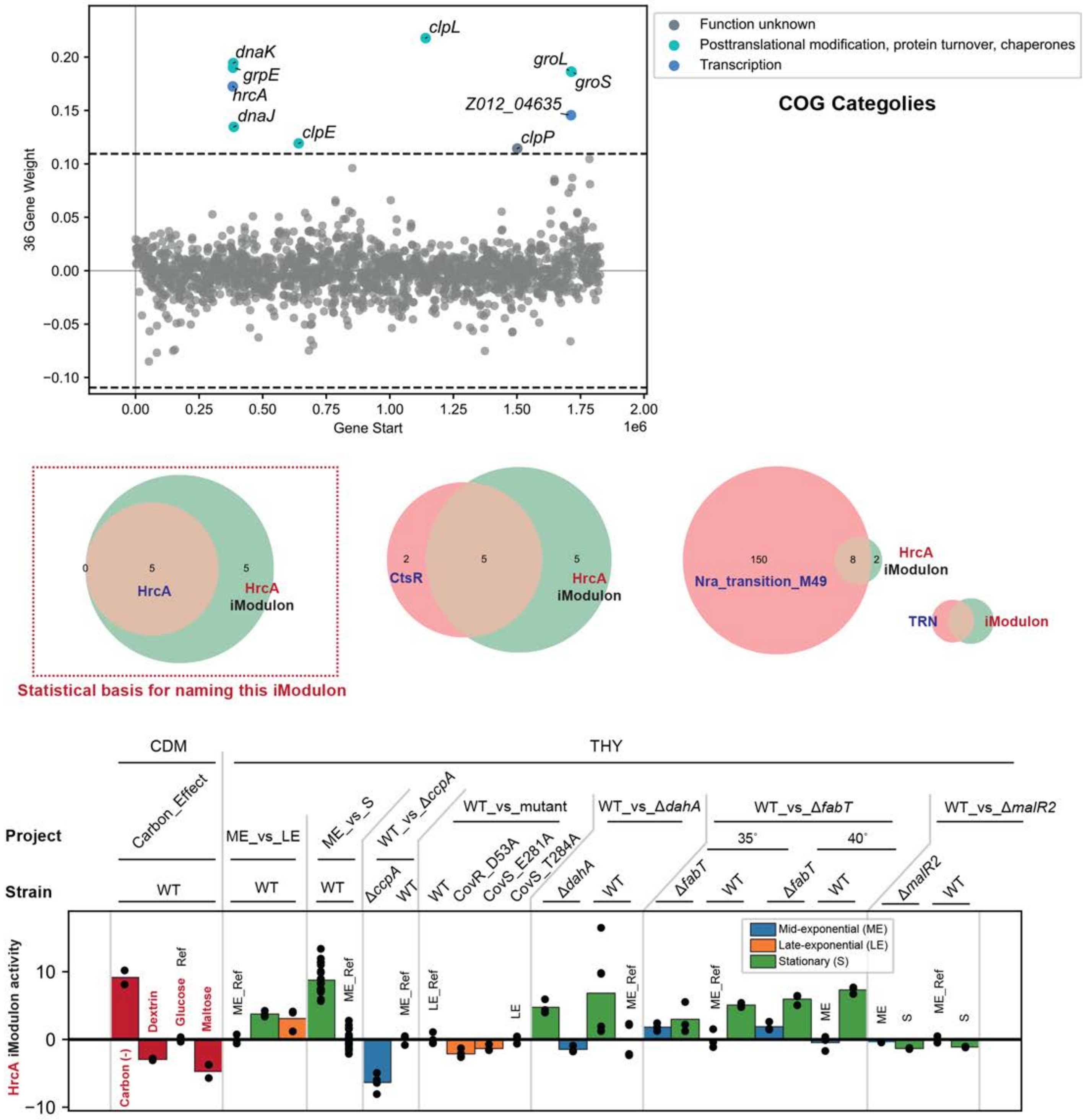

Phage_3-2 iModulon

Significantly overlapped TRN: Phage_3 > Phage_1

Biological function: Prophages

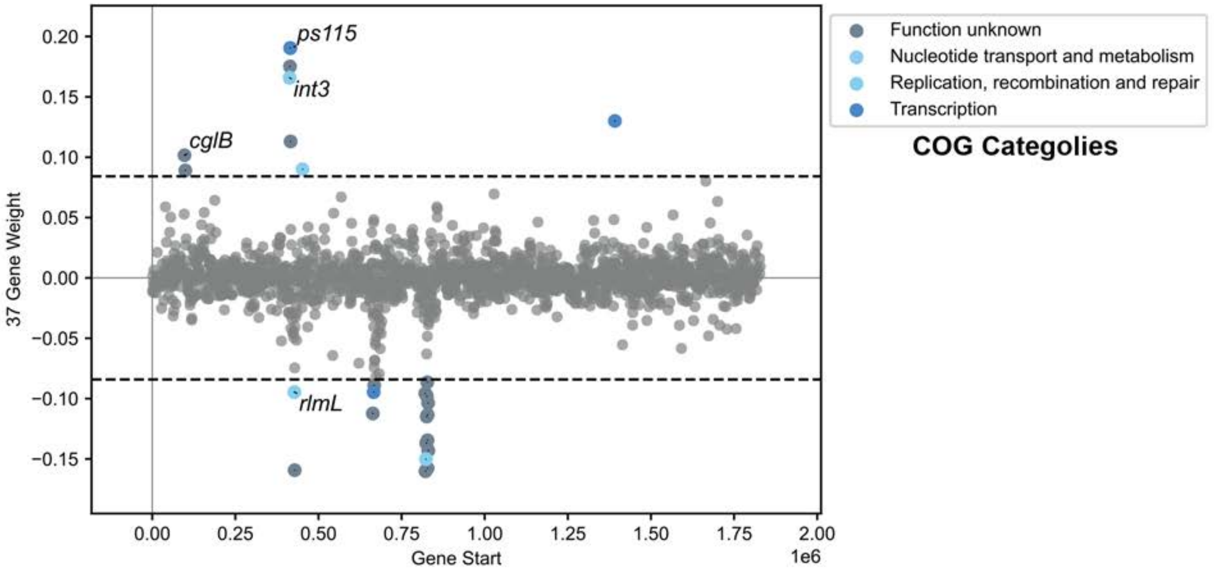

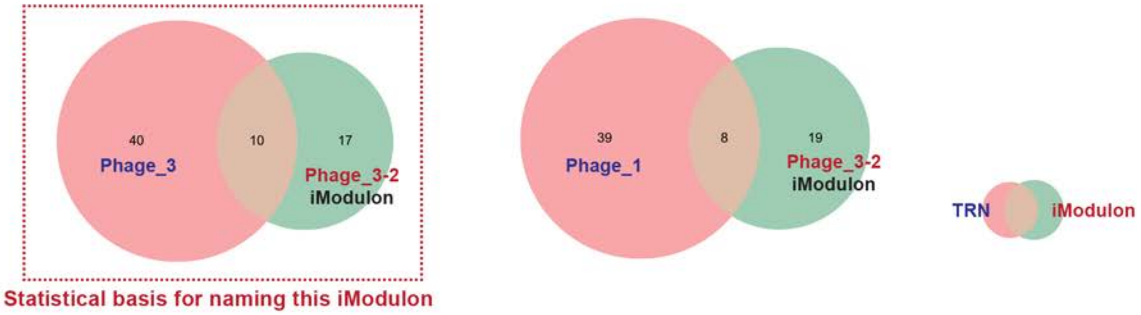

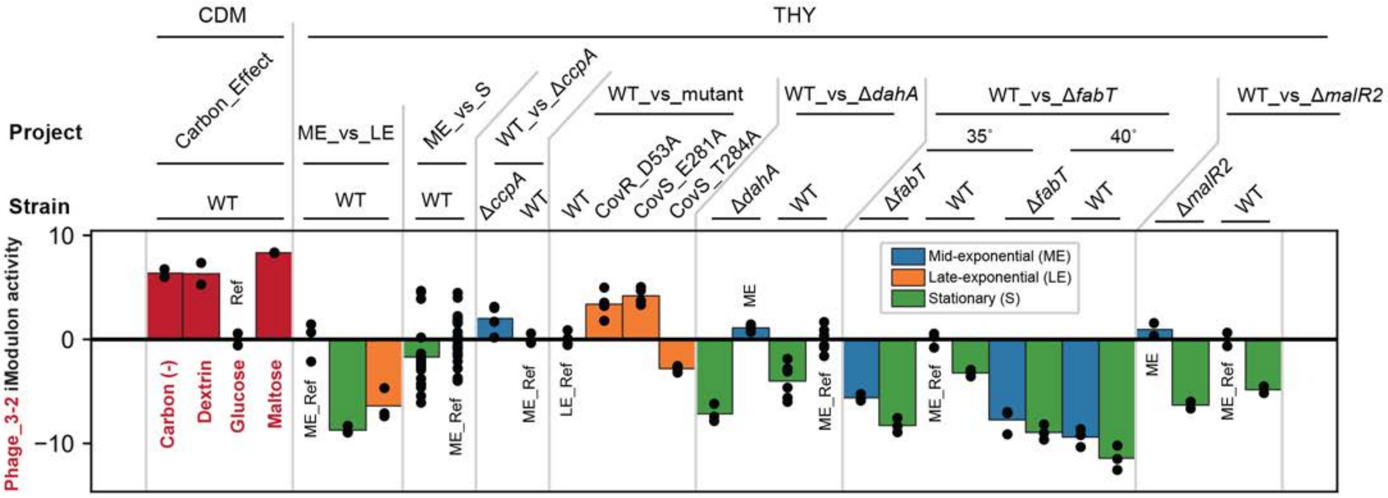

SpeB iModulon

Significantly overlapped TRN: ---

Biological function: Toxin production

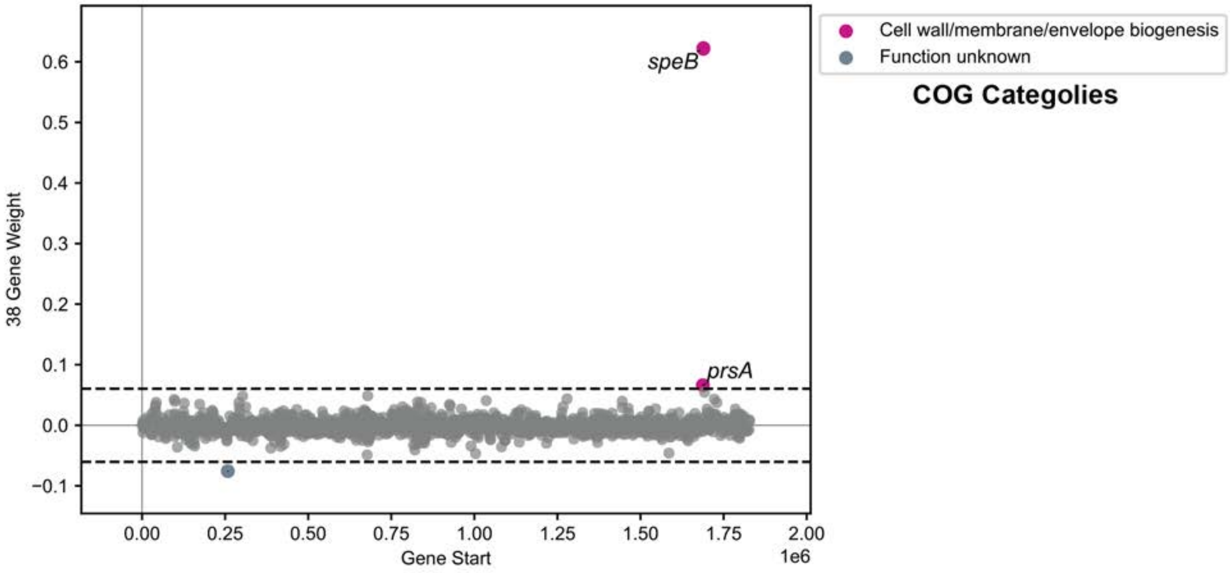

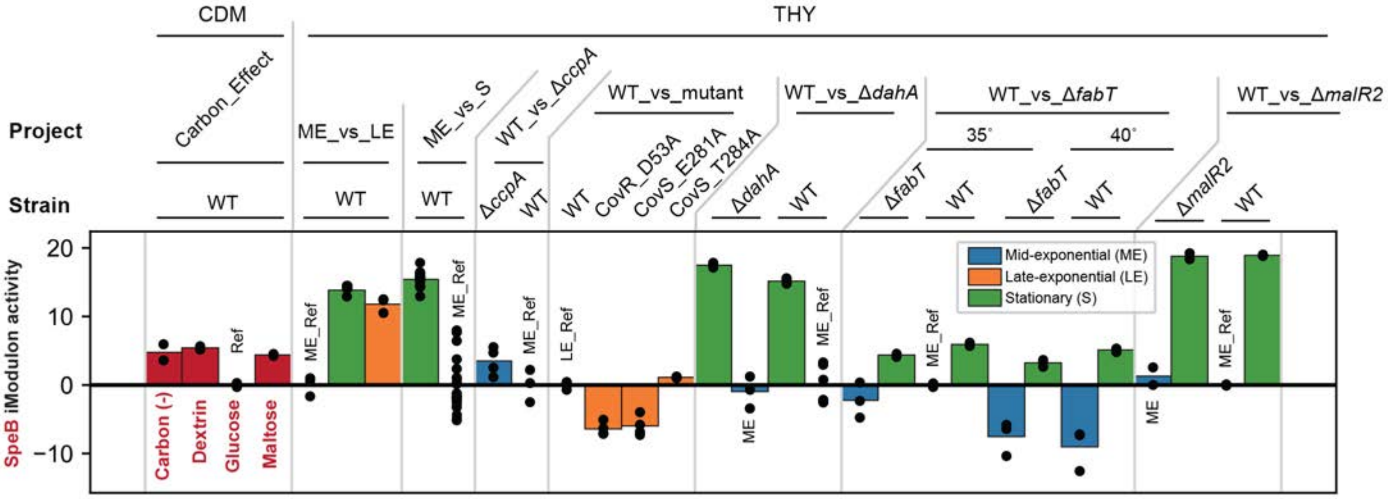

Transport-2 iModulon

Significantly overlapped TRN:

Biological function: Multi-purpose metabolism

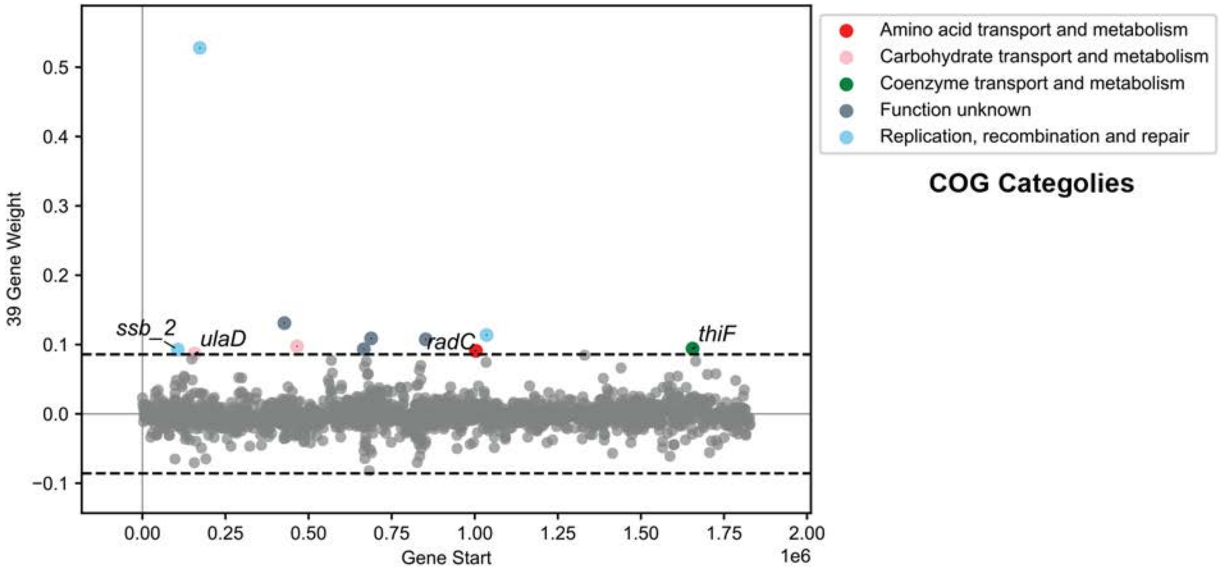

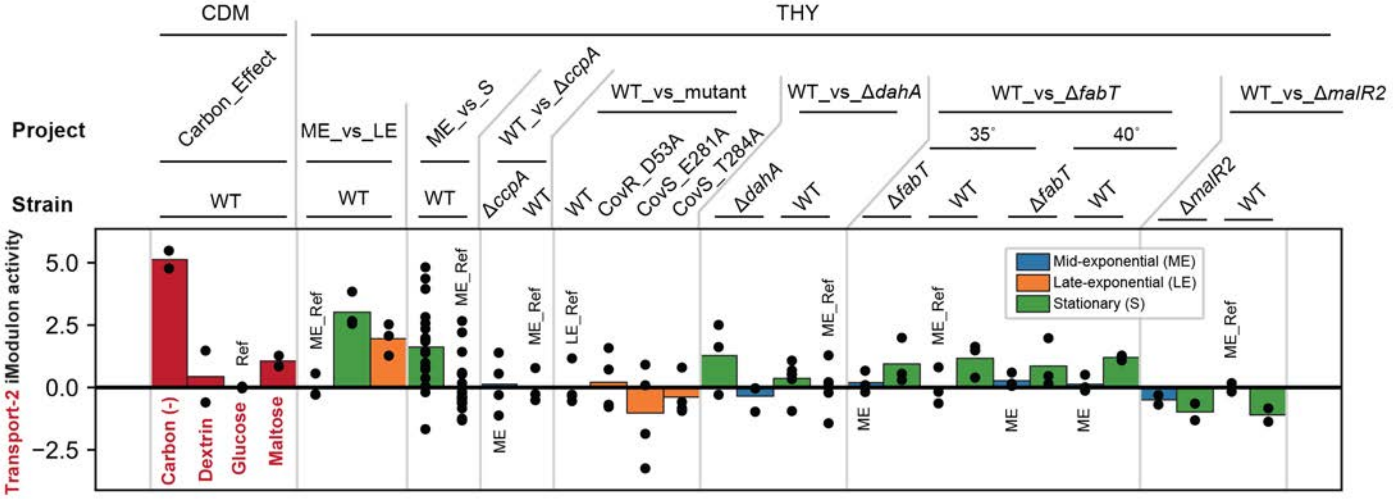

Unc_12 iModulon

Significantly overlapped TRN: ---

Biological function: General stress

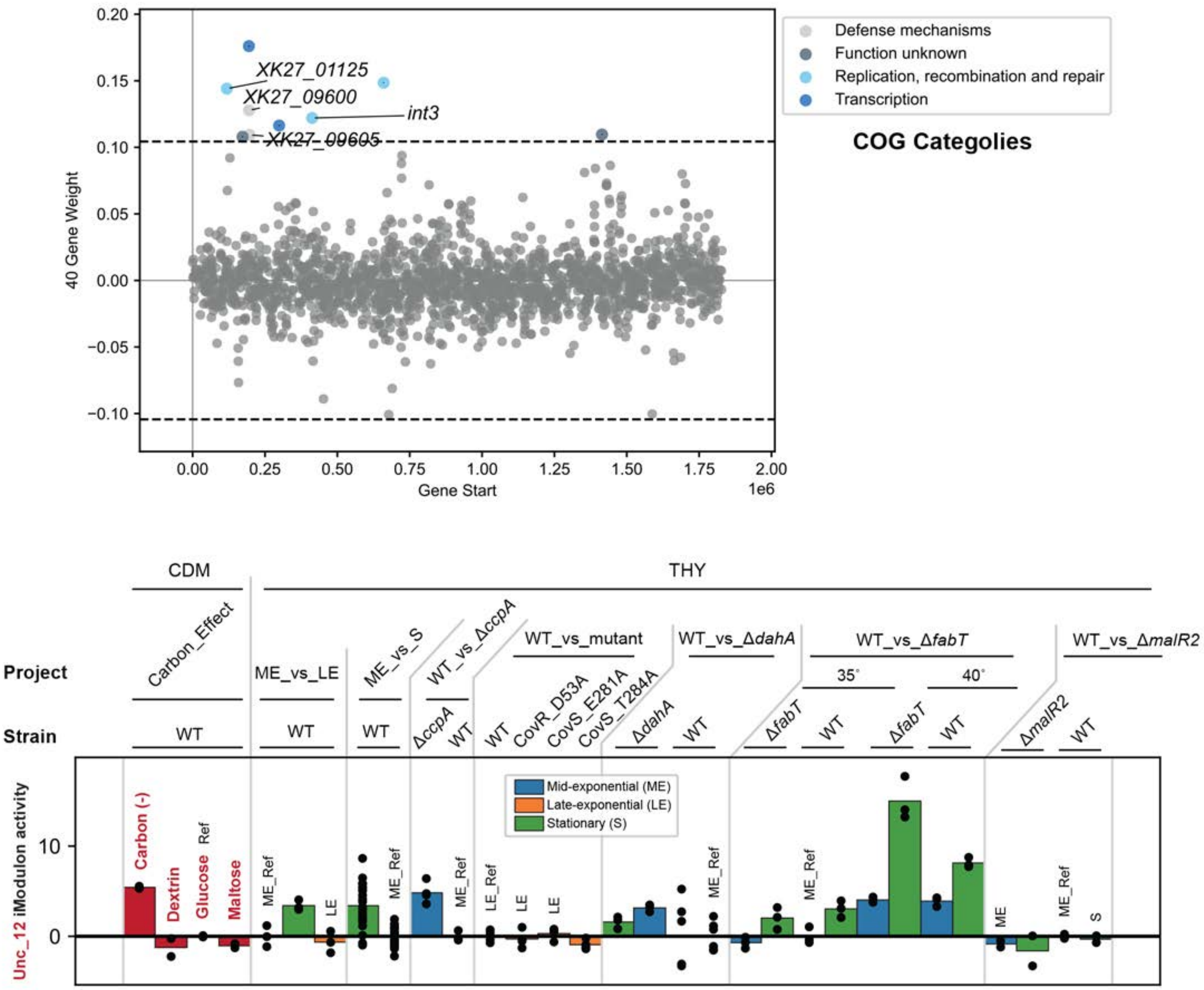

SLO iModulon

Significantly overlapped TRN: ---

Biological function: Toxin production

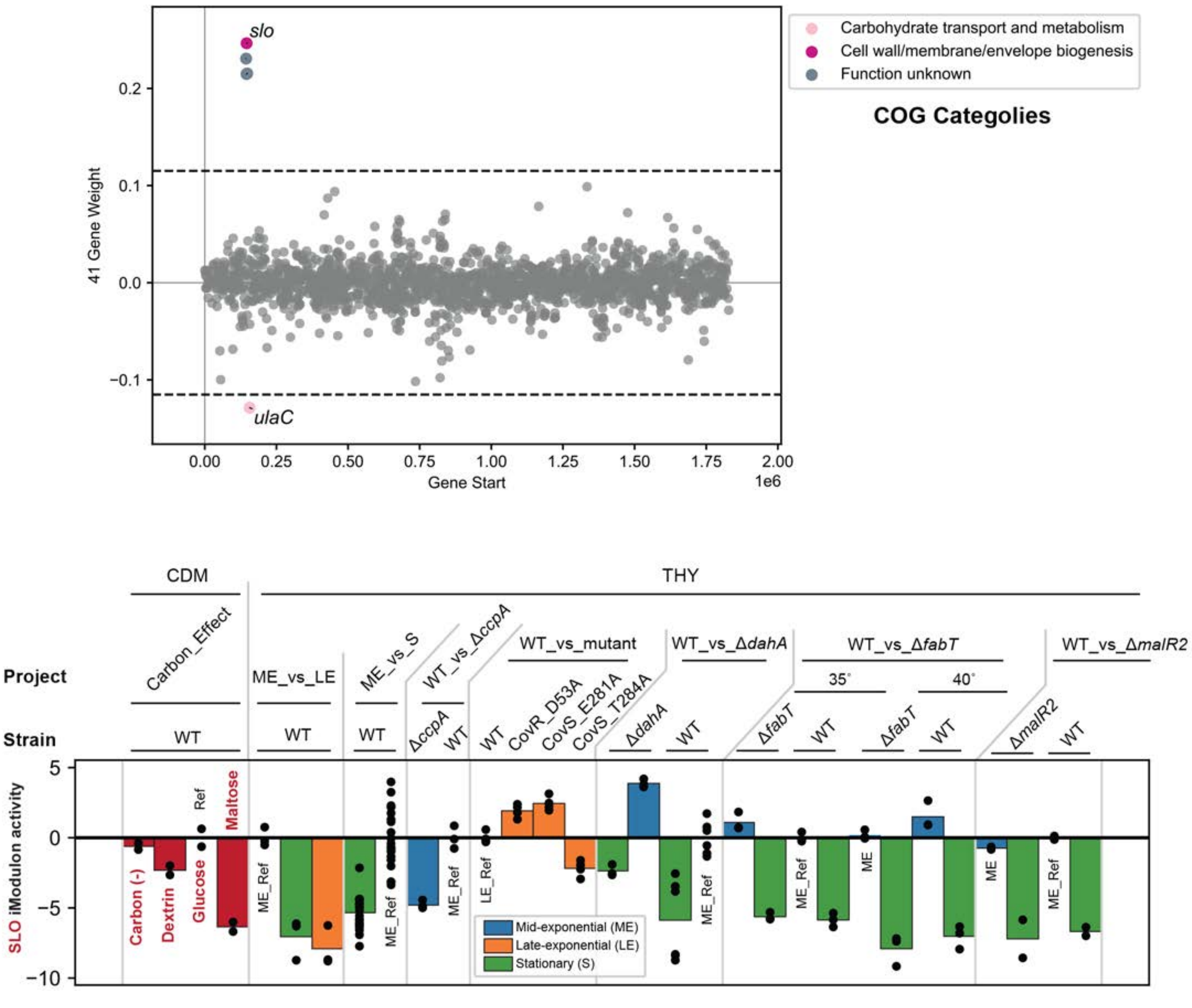

Supplement: Supplementary information 1 — Graphical representation of 42 iModulons. [file msystems.00247-23-s0004.pdf]
